# Integrative biochemical, proteomics and metabolomics cerebrospinal fluid biomarkers predict clinical conversion to multiple sclerosis

**DOI:** 10.1093/braincomms/fcab084

**Published:** 2021-04-19

**Authors:** Fay Probert, Tianrong Yeo, Yifan Zhou, Megan Sealey, Siddharth Arora, Jacqueline Palace, Timothy D W Claridge, Rainer Hillenbrand, Johanna Oechtering, David Leppert, Jens Kuhle, Daniel C Anthony

**Affiliations:** 1 Department of Pharmacology, University of Oxford, Oxford OX1 3QT, UK; 2 Department of Chemistry, University of Oxford, Oxford OX1 3TA, UK; 3 Department of Neurology, National Neuroscience Institute, Singapore 308437, Singapore; 4 Mathematical Institute, University of Oxford, Oxford OX2 6GG, UK; 5 Nuffield Department of Clinical Neurosciences, John Radcliffe Hospital, University of Oxford, Oxford OX3 9DU, UK; 6 Novartis Pharma AG, Basel CH-4056, Switzerland; 7 Neurology, Departments of Medicine, Clinical Research and Biomedicine, University Hospital Basel, University of Basel, Basel CH-4031, Switzerland

**Keywords:** multiple sclerosis, clinically isolated syndrome, biomarker, prediction, prognosis

## Abstract

Eighty-five percent of multiple sclerosis cases begin with a discrete attack termed clinically isolated syndrome, but 37% of clinically isolated syndrome patients do not experience a relapse within 20 years of onset. Thus, the identification of biomarkers able to differentiate between individuals who are most likely to have a second clinical attack from those who remain in the clinically isolated syndrome stage is essential to apply a personalized medicine approach. We sought to identify biomarkers from biochemical, metabolic and proteomic screens that predict clinically defined conversion from clinically isolated syndrome to multiple sclerosis and generate a multi-omics-based algorithm with higher prognostic accuracy than any currently available test. An integrative multi-variate approach was applied to the analysis of cerebrospinal fluid samples taken from 54 individuals at the point of clinically isolated syndrome with 2–10 years of subsequent follow-up enabling stratification into clinical converters and non-converters. Leukocyte counts were significantly elevated at onset in the clinical converters and predict the occurrence of a second attack with 70% accuracy. Myo-inositol levels were significantly increased in clinical converters while glucose levels were decreased, predicting transition to multiple sclerosis with accuracies of 72% and 63%, respectively. Proteomics analysis identified 89 novel gene products related to conversion. The identified biochemical and protein biomarkers were combined to produce an algorithm with predictive accuracy of 83% for the transition to clinically defined multiple sclerosis, outperforming any individual biomarker in isolation including oligoclonal bands. The identified protein biomarkers are consistent with an exaggerated immune response, perturbed energy metabolism and multiple sclerosis pathology in the clinical converter group. The new biomarkers presented provide novel insight into the molecular pathways promoting disease while the multi-omics algorithm provides a means to more accurately predict whether an individual is likely to convert to clinically defined multiple sclerosis.

## Teaser

Probert et al. use an integrative analysis approach to identify novel CSF biomarkers from biochemical, proteomics, and metabolomics assays for the prediction of clinically conversion to multiple sclerosis in patients recruited at clinically isolated syndrome onset. The proposed multivariate algorithm significantly outperforms oligoclonal band status for prediction of clinical conversion.

## Graphical Abstract

**Graphical Abstract:**
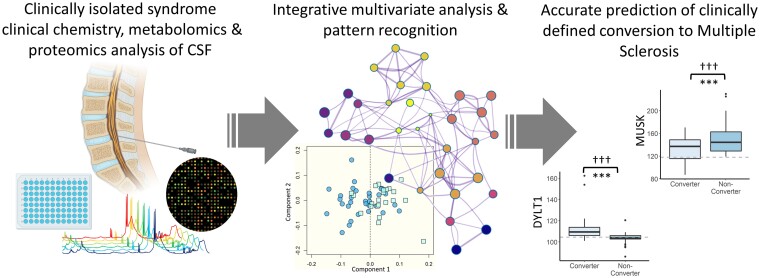

## Introduction

Clinically isolated syndrome (CIS) is the first manifestation of multiple sclerosis (MS) in 85% of patients.^1^ However, not all patients with a CIS attack go on to a confirmed MS diagnosis. Indeed, 37% of CIS patients do not fulfil McDonald 2010 diagnostic criteria 20 years after onset^2^ while only 63% transition to clinically defined MS, which is, in practice, the occurrence of a second clinical attack.^3^ Early treatment is essential to minimize the occurrence of further attacks and the accumulation of permanent disability.^4–9^ Thus, differentiation of individuals who are most likely to have a second clinical attack from those who remain in the CIS stage is essential to achieve the desired personalized medicine approach.

Currently, MS diagnosis relies upon exclusion of other possible diagnoses followed the interpretation of a combination of detailed clinical evaluation, magnetic resonance imaging (MRI), and CSF analysis according to the latest McDonald Diagnostic criteria.^10^ The revisions introduced in the 2017 McDonald criteria [namely the inclusion of CSF oligoclonal IgG bands (OCGB) as a surrogate maker for dissemination in time] have resulted in more patients being diagnosed with MS at the point of CIS, this is at the expense of reduced specificity of only 61%–63% compared to 85%^11^^,^^12^ using the previous 2010 criteria. Hence, these revisions undoubtedly benefit patients by ensuring that MS is treated in timely manner. However, the McDonald criteria are not meant to predict the course for disability worsening or time to second relapse.

Recognised risk factors of clinically defined conversion from CIS to MS have been identified including younger age of disease onset, male gender,^13^^,^^14^ the number of T_2_ weighted MRI lesions,^15^ the presence of OCGB,^16^ intrathecal IgM synthesis^17^^,^^18^ and neurofilament light chain.^19^ However, these measures are validated only at the group level, and there is no biofluid or imaging marker to address this unmet need for individual use. For example, 21% of CIS patients with normal MRI scans at baseline still transition to clinically defined MS and, while OCGB are extremely sensitive for the diagnosis of MS, 50% of CIS patients who test positive for OCGB at baseline do not have a second clinical attack within 50–60 months.^16^^,^^19^^,^^20^

To identify novel predictive biomarkers for the transition to MS we collected baseline CSF samples from 54 patients with CIS with 2–10 years of follow-up to determine clinical conversion. This study represents the first comprehensive multi-omics investigation of CIS to include simultaneously clinical chemistry, metabolomics and proteomics analysis of baseline CSF samples coupled with extensive clinical follow-up and to investigate the power of such biomarkers at predicting clinically defined conversion in an individualized manner. Here we report the capacity of novel biochemical and protein markers to predict the transition from CIS to clinically defined MS. The future use of simple multivariant biomarker algorithms in the clinical care pathway has the evident potential to facilitate the personalization and optimization of therapy for individuals with CIS.

## Materials and methods

### Study participants

CSF samples were collected in the Department of Neurology of the University Hospital Basel, during routine diagnostic measures, as indicated by the treating physicians. Inclusion criteria were as follows: (i) the presence of a monophasic clinical episode suggestive of MS (CIS), not attributable to other diseases (for example, infectious, neoplastic, congenital, metabolic or vascular disease)^21^; (ii) clinical follow-up of at least two years; (iii) available basic demographic and clinical data (age, gender, dates of CIS onset, serum sampling, CSF examination, MRI, clinically defined conversion to MS (if present) and last follow-up visit); and (iv) CSF information at time of CIS. Patients with neuromyelitis optica, or a history of a progressive disease course from onset were excluded. Exclusion criteria included (i) active systemic infection and (ii) steroid treatment at the time of CSF sampling. Conversion to MS was diagnosed according to Poser criteria. This implied the exclusion of alternative diagnoses and the presence of a second clinically evident demyelinating attack which had to be separated in time and space from the first episode (i.e. occurring after an interval of at least one month and in a separate CNS location).^22^ In total, 85 individuals were recruited at CIS onset and examined for eligibility in the study. Of these, three were confirmed to have active infections and two were receiving steroid treatment at the point of CSF sampling while 26 had insufficient follow-up to determine clinical converter status and were excluded from the study. A flow chart of sample recruitment and exclusions can be found in Supplementary Fig.1. There were no missing data for any of the 54 participants included in the analysis. CIS patients were recruited based on the criteria above to avoid bias and no significant difference in age, gender or onset expanded disability scale status (EDSS) was observed between the converter and non-converter groups.

### Standard protocol approvals, registrations and patient consents

Written informed consent was obtained from all patients according to the Declaration of Helsinki. Ethical approval was obtained by the local ethics committee.

### Power calculation

Prior to analysis, a power calculation (PPCA model using the R package MetSizeR) was carried out. This confirmed that a sample size of 40 (20 non-converters and 20 converters) would be sufficient to achieve an FDR cut-off of 0.05 assuming the significance of 10% of variables. These assumptions are in line with our previous omics analysis of MS patient cohorts and indicated that *n* = 22 in the converter group would be sufficient.

### CSF sample collection

CSF samples were centrifuged at 400 *g* for 10 min at room temperature, and the cell-free supernatant stored at −80°C within 2 h of collection.^23^ Samples were processed as per standard laboratory procedures for leukocytes (cells/mm^3^), and total protein concentration (mg/dl). Serum samples were collected at the same visit to calculate the CSF/serum albumin ratio (Q_alb_). The integrity of the blood–CSF barrier was determined by calculating the CSF/serum ratio for albumin (Q_alb_).^24^ Intrathecal synthesis of IgG was determined by detection of oligoclonal IgG bands (OCGB) by isoelectric focussing on agarose gel and subsequent immunoblotting using IgG-specific antibody staining.^25^ Testing of OCGB was considered positive if pattern two or three (local synthesis of IgG within the CNS) were present.^26^ These parameters are henceforth referred to as clinical chemistry parameters.

### Nuclear magnetic resonance sample preparation for metabolomics analysis

On the day of metabolomics analysis, CSF samples were thawed at room temperature and 100 µl was then diluted with 450 μl of 75 mM sodium phosphate buffer prepared in D_2_O (pH 7.4) containing 1 mM maleic acid as an internal reference standard. Samples were briefly centrifuged at 3000 × *g* for 5 min before transferring to a 5-mm NMR tube.

### Nuclear magnetic resonance spectroscopy and data processing for metabolomics analysis

All nuclear magnetic resonance (NMR) spectra were acquired at 310 K using a 700-MHz Bruker AVIII spectrometer operating at 16.4 T equipped with a ^1^H [^13^C/^15^N] TCI cryoprobe (Department of Chemistry, University of Oxford). The noesygppr1d (Bruker, Germany) pulse sequence was used to acquire ^1^H NMR spectra with a 2 s presaturation, 32 data collections, a spectral width of 16 ppm, and an acquisition time of 1.46 s. All spectra were preprocessed in Topspin 2.1 (Bruker, Germany); zero filled by a factor of 2 and multiplied by a 1D exponential corresponding to a 0.3 Hz line broadening. All spectra were baseline corrected with a fifth-degree polynomial and referenced to the lactate doublet at 1.33 ppm. Following visual inspection for errors in baseline correction, referencing, spectral distortion or contamination, the processed spectra were exported to ACD/Labs Spectrus Processor Academic Edition 12.01 (Advanced Chemistry Development, Inc., Toronto, Canada), whereby regions of the spectra between 0.83 and 8.47 were split into 0.02-ppm-wide bins. The residual water resonance region (4.13–5.22 ppm) was removed from the analysis. The integral of each spectral bin was calculated and exported as a .csv file for statistical analysis. Metabolite assignment was performed by referencing to literature values,^27^^,^^28^ the Human Metabolome Database^29^ and via 2 D correlation spectroscopy (COSY) experiments. A list of all NMR-detectable CSF metabolites has been previously reported.^30^ While, all metabolite resonances were included in our analysis the 30 most abundant metabolites detectable in the NMR spectra included (in alphabetical order) 3-hydroxybutyrate, acetate, acetoacetate, alanine, arginine, aspartate, citrate, creatine, creatinine, formate, glucose, glutamate, glutamine, glycerol, histidine, isoleucine, lactate, leucine, lysine, methyl isobutyrate, myo-inositol, N-acetyl-aspartate, phenylalanine, proline, scyllo-inositol, taurine, threonine, trimethyl-amine, tyrosine and valine.

### Determining concentration of the NMR metabolite biomarkers

NMR metabolite measures, in relative units, were converted to absolute concentrations in SI units using an internal reference standard (1 mM maleic acid). In order to validate the quantification of the metabolites by NMR, the glucose and lactate levels in all CSF samples were measured using a Cobas^®^ 8000 modular analyzer (Roche Diagnostics, Switzerland) and the Gluc3 and LAC2 assays, respectively. There was a significant correlation between the NMR-determined concentration and the laboratory chemistry determined concentration for both glucose (Pearson’s *R* = 0.91, *P*-value < 0.001) and lactate (Pearson’s *R* = 0.90, *P*-value < 0.001) (Supplementary Fig. 4Aand B) and Bland–Altman plots revealed excellent agreement between the two methods (Supplementary Fig. 4Cand D).

### Protein profiling by SomaScan^TM^

Protein biomarker profiling in CSF samples was performed using the SomaScan^®^ platform from SomaLogic Somalogic Inc, Boulder, Co).

SomaScan^®^ is multiplexed proteomic tool that measures more than 5000 protein analytes including 4783 SOMAmers (slow off-rate modified aptamers) that recognize 4137 distinct human gene targets. The SOMAmers are constructed with chemically modified nucleotides that expand the physicochemical diversity of the large randomized nucleic acid libraries from which the SOMAmer reagents are selected. The SomaScan^®^ assay measures native proteins in complex matrices by transforming each individual protein concentration into a corresponding SOMAmer reagent concentration, which is then quantified in customized DNA microarrays. The assay takes advantage of SOMAmer reagents’ dual nature as both protein affinity-binding reagents with defined three-dimensional structures, and unique nucleotide sequences recognizable by specific DNA hybridization probes.^31^^,^^32^

The CSF samples were stored at −80°C and shipped to SomaLogic (Boulder, CO) on dry ice for SomaScan^®^ analysis.

### Metabolomics & proteomics statistical analysis

Multivariate orthogonal partial least squares discriminant analysis (OPLS-DA) was performed in R software (R foundation for statistical computing, Vienna, Austria)^33^ using in-house R scripts and the ropls package.^28^ OPLS-DA is a dimension reduction technique that can extract correlated patterns from complex data sets. This method is ideally suited for large *‘*omics datasets (as used in other similar aforementioned studies), such as those studied here, because it helps reduce the dimension of such datasets by extracting a subset of most salient biomarkers (described as a linear equation) that can subsequently be used to predict the class of interest—in this instance clinical converters and non-converters. OPLS-DA models were validated using an external 10-fold cross-validation strategy with repetition coupled with permutation testing as previously described.^34^ We have previously published an in-depth description of this analysis approach^35^ and further detail can be found in the Supplementary material. In brief, the data are corrected for unequal class sizes before being randomly split into a training set (90%) and an independent test set (10%). The training set is used to build the OPLS-DA model the *R*^2^ and *Q*^2^ values are then used to assess the model performance (the goodness of fit and prediction, respectively) on the training data. The model generated is then applied to the test set (to which the OPLS-DA model is blinded) to determine the predictive accuracy, sensitivity, and specificity of the model (on previously, unseen data). This process of model training and testing is repeated for a total of 1000 times, thereby creating an ensemble of models. It has been shown that in cases where the sample sizes are small, one can achieve a prediction accuracy of up to 70% or higher by chance alone in differentiating two-classes.^36^ Thus, to further validate our prediction accuracy, we compare our ensemble of models to an ensemble of randomly permuted models (generated by randomly permuting the class identities). For a 2-class classification problem, the expected accuracy of a randomly permuted model is 50%. If the model ensemble significantly outperforms the randomly permuted models (as quantified using a two-sided Kolmogorov–Smirnov test, significant if *P*-value 0.05 or less), then the discriminatory variables responsible for the observed class separation are extracted by inspection of the average variable importance (VIP) scores. The VIP score of a given variable represents the mean decrease in accuracy which occurs when that variable is removed from the model. Thus, a variable which is highly significant and plays a large role in the diagnostic accuracy of the model will result in a large decrease in accuracy when removed from the model resulting a large VIP score. Conversely, variables which do not play a role in discriminating between groups have very little effect on model accuracy when removed and have a low VIP score.

Elastic Net feature selection was applied to the proteomics data using the glmnet package^37^ prior to each iteration of the OPLS-DA method to reduce the number of predictor variables (by removing irrelevant or redundant variables), which helps improve model inference and lowers computational time.^38^^,^^39^ Both the α and λ for use in each elastic net feature selection were determined using 7-fold cross validation to optimize the mean squared error on the training data alone.

In order to identify the combination of clinical chemistry, metabolomics and proteomics variables with the highest predictive accuracy a combined multi-omics strategy was applied to the selected discriminatory variables (all variables from the clinical chemistry, proteomics, and metabolomics data combined) by applying the OPLS-DA cross-validation strategy (described above) to every combination of one to six variables and performing a ROC analysis on each model to assess the performance. The linear combination of metabolites which resulted in the highest performing model (determined by AUC, accuracy, sensitivity and specificity) are then reported. There was no significant increase in AUC or accuracy between five and six variables and so linear combinations of variables containing greater than 6 were not pursued.

### Univariate and ROC analysis

All analysis was performed in R software (R foundation for statistical computing, Vienna, Austria). Two-sample *t*-tests were used for continuous variables while Chi-square tests were used for categorical variables as appropriate. A Bonferroni correction, to account for multiple comparisons, was applied throughout. Two-tailed *P*-values < 0.05 were considered statistically significant. Receiver operator curves (ROC), area under the curve (AUC), 95% confidence intervals, optimal thresholds for diagnosis and *P*-values (relative to a null distribution ROC curve with AUC = 0.5) were calculated for each discriminatory variable using the pROC package.^40^ The diagnostic odds ratio for each biomarker identified is reported. Where one group in the contingency was empty the Haldane–Anscombe correction was used.^41^

### Protein pathway analysis

Pathway enrichment analysis was performed on the discriminatory proteins identified by the multivariate analysis (described above) using Metascape [http://metascape.org].^42^ Metascape is updated monthly and combines over 40 independent knowledgebases including GO, KEGG and MSigDB for enrichment and gene membership analysis. All genes in the genome were used as the enrichment background. Terms with a *P*-value < 0.01, a minimum count of 3 and an enrichment factor (the ratio of observed counts to the counts expected by chance) >1.5 were collected and grouped into clusters based on their membership similarities. Kappa scores^43^ were used to define the extent of ‘similarity’ when performing hierarchical clustering on the enriched terms, and sub-trees with a similarity of >0.3 are considered a cluster. The most statistically significant term within any given cluster was chosen to represent that cluster. Cytoscape was used to visualize the results of the protein enrichment network.^44^ The DisGeNet discovery platform^45^ (www.disgenet.org last accessed 27/04/2021) and Enrichr^46^ (https://maayanlab.cloud/Enrichr/ last accessed 27/04/21) was used to perform enrichment analysis on the identified proteins which were up/down regulated in the converter cohort relative to the non-converter cohort in an effort to identify disease specific pathways associations.

### Data and code availability

Anonymized data and code will be shared by request from any qualified investigator.

## Results

### While useful in diagnosis, baseline OCGB positivity is unable to predict clinically defined conversion

A total of 54 patients with symptoms consistent with CIS were included in this study. CSF samples were collected within one year of CIS onset [mean time to sample collection from onset 3.5 (1–11) months] and followed up for up to 10 years. Twenty-two patients converted to clinically defined MS (hence forth referred to as ‘converters’) while 32 patients had no signs of further relapses during follow-up (hence forth referred to as ‘non-converters’). The median time to sample collection in both the converter and non-converter groups was 3 weeks and there was no significant difference in the means (*P*-value 0.82). All converter CSF samples were collected before the second attack. Patient demographics and clinical chemistry results for both converters and non-converters are reported in Table 1. Length of follow-up was longer in the non-converter group [mean, 6.5 years; median, 7.1 years; range, 2.0–9.7 years; interquartile range (IQR), 5.6–8.1 years] relative to the converter group (mean, 4.6 years; median, 4.2 years; range 1.6–8.7 years; IQR, 3.1–5.3 years) to ensure that sufficient time elapsed for relapses to occur. As the majority of CIS patients experience a second attack within two years of onset with the median and mean time to second attack ranging from 11–14 months and 8–11 months, respectively,^13^^,^^20^^,^^47^^,^^48^ patients were only included in the non-converter group if a minimum of two years of follow-up was available. Indeed, the average time to conversion in the converter group was 1.7 years (median, 0.9 years; IQR, 0.4–2.1 years). Thus, the length of follow-up in the non-converter group (median 7.1 years) was significantly greater than the time to conversion in the converter group (median 0.9 years), suggesting the length of follow up in the non-converters is sufficient. There were no significant differences in the gender distributions, age, or grade of disability as per EDSS score at onset between the converter and non-converter groups.

**Table 1:** Patient demographic and clinical data, at the point of CSF sampling, grouped by converter status.

	Converter [*n* = 22]	Non-converter [*n* = 32]	*P*-value
Female, No. [%]	17 [77]	21 [66]	0.36
Age, mean [SD], years	31.3 [9.9]	36.4 [11.2]	0.08
EDSS, median [range]	2.5 [0–4]	1.5 [0–4]	0.06
Time to conversion, mean [SD], years	1.7 [2.0]	NA	NA
Follow-up, median [IQR], years	4.2 [3.1–5.3]	7.1 [6.0–8.1]	<0.001
Immune modulating therapies	None	None	NA
OCGB positive, No. [%]	22 [100]	22 [69]	<0.001
Leukocytes, mean [SD], cells/mm^3^	10.9 [9.1]	4.9 [4.9]	<0.001
Mononuclear, mean [SD], cells/mm^3^	10.7 [8.8]	4.7 [4.7]	<0.001
Polynuclear, mean [SD], cells/mm^3^	0.27 [0.65]	0.21 [0.64]	0.73
Total protein, mean [SD], mg/dl	3367.7 [96.0]	374.7 [96.0]	0.83
CSF/serum albumin ratio, mean [SD]	4.9 [2.0]	5.2 [1.6]	0.58

*P*-values from Student’s *t*-test for continuous variables and Chi-squared test for categorical variables are reported.

EDSS, expanded disability status scale; IQR, interquartile range; OCGB, oligoclonal bands; SD, standard deviation.

**Figure 1:**
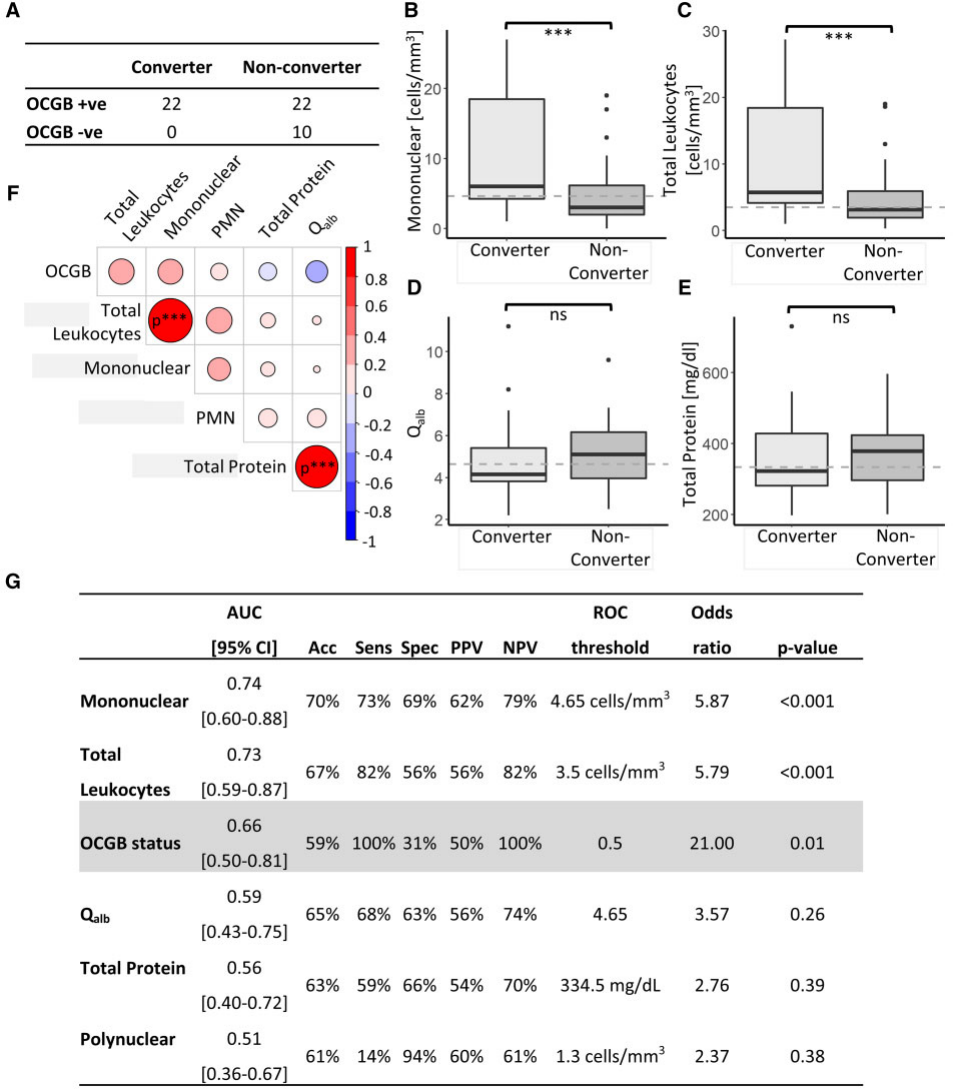
**Clinical chemistry results.** (**A**) Confusion matrix illustrating the low sensitivity of OCGB status for the prediction of clinically defined conversion to MS. Box plots of CSF clinical chemistry parameters (**B**) mononuclear cells, (**C**) leukocyte cells, (**D**) CSF/serum albumin ratio (Q_alb_) and (**E**) total protein measured in clinically defined converters and non-converters. Dashed lines represent the optimal threshold to achieve the greatest accuracy as determined by ROC analysis. A significant increase in mononuclear and leukocytes cell counts was observed in the converter group. (**F**) Heat map illustrating all correlations (Pearson’s *R*) between the CSF clinical chemistry measures and OCGB. (**G**) Predictive performance of CSF clinical chemistry parameters compared to the performance of OCGB status. Biomarkers are listed from highest to lowest AUC. Acc, Accuracy; AUC, area under the curve; CI, confidence interval; NPV, negative predictive value; PPV, positive predictive value; ROC, receiver operator curve; Sens, sensitivity; Spec, specificity. Clinically defined converter *n* = 22, clinically defined non-converter *n* = 32. Two-sample *t*-tests were used for continuous variables while Chi-square tests were used for categorical variables as appropriate. A Bonferroni correction to account for multiple comparisons was applied throughout. Two-tailed *P*-values <0.05 were considered statistically significant. *P*-values <0.001 following correction for multiple comparisons are represented by ***.

While useful in the diagnosis of MS in the context of the revised McDonald criteria, the presence/absence of OCGB was not predictive of transition to clinically defined MS. Although more of the converters tested positive for OCGB (100%) still the majority (69%) of the non-converters also tested positive at onset (Fig. 1A). As a result, OCGB are extremely sensitive (100%) for the prediction of a second clinical attack, but specificity is very low (31%) resulting in an AUC and accuracy of only 0.66 and 59%, respectively. It should be noted that the average length of follow-up in the OCGB+ve non-converters (mean, 4.6 years; median, 4.2 years; range, 2–10 years; IQR, 3.1–5.3 years) was, again, significantly greater than the median time to conversion (0.9 years) in the converter group, indicating that the absence of a second attack in these OCGB+ve was not a result of follow-up duration.

### Baseline CSF leukocyte and mononuclear cell counts predict clinically defined conversion with greater overall accuracy than OCGB status

In addition to OCGB, several clinical chemistry parameters were measured at CIS onset including leukocyte [further divided into mononuclear and polymorphonuclear (PMN) cell counts], total CSF protein levels and Q_alb_. There was no significant difference in the Q_alb_ or total CSF protein concentration between converters and non-converters at onset. Twenty-nine patients (54%) had ‘normal’ (< 4 cell/mm^3^) CSF leukocyte cell counts at baseline. The highest leukocyte cell count was 28.7 cell/mm^3^ and 13 (24%) patients (nine converters and four non-converters) exhibiting a cell count above 10 cell/mm^3^ at onset. The leukocyte cell count was increased (>4 cell/mm^3^) in a larger proportion of converter patients (72%) than non-converters (41%) (Chi-Squared *P*-value 0.02). Interestingly, the mononuclear cell sub-population was elevated in the converter group relative to non-converters, while the PMN subset was not significantly altered (Fig. 1B–E). Indeed, ROC analysis reveals that the CSF leukocyte cell count and, particularly, the monocular cell population outperform OCGB for the prediction of a second clinical attack with AUC values of 0.74 and 0.73, respectively (Fig. 1G). Mononuclear cell counts were strongly correlated with leukocyte levels (Pearson’s *r* 0.96, *P*-value < 0.001 corrected for multiple comparisons) and weekly correlated with PMN although this correlation did not reach significance following correction for multiple comparisons (Pearson’s *r* 0.35, *P*-value 0.12 corrected for multiple comparisons). As expected, a strong correlation (Pearson’s *r* 0.94, *P*-value < 0.001 corrected for multiple comparisons) between total protein levels and Q_alb_ was observed (Fig. 1F).

### Myo-inositol and glucose CSF concentrations outperform OCGB status for prediction of clinical conversion to MS

**Figure 2:**
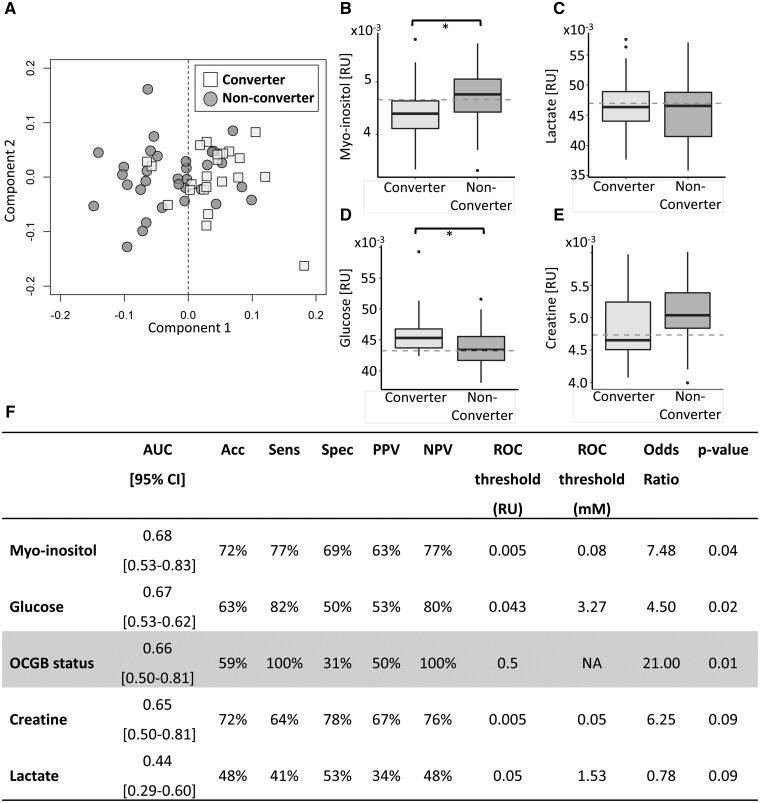
**Metabolomics results.** (**A**) Representative OPLS-DA scores plot illustrating discrimination between clinically defined converters (square) and non-converters (circle) CSF metabolite profiles. (**B–E**) Boxplots of the significant discriminatory metabolites identified by the OPLS-DA analysis in clinically defined converters and non-converters. Dashed lines represent the optimal threshold to achieve the greatest accuracy as determined by ROC analysis. For univariate analysis, two-sample *t*-tests were used for continuous variables while Chi-square tests were used for categorical variables as appropriate. While for multivariate analysis a two-sided Kolmogorov–Smirnov test was used to determine the significance of the OPLS-DA performance on independent test data relative to the null distribution. A Bonferroni correction to account for multiple comparisons was applied throughout. Univariate *P*-values below 0.05, 0.01 and 0.001 are represented by *, ** and ***, respectively. (**F**) Predictive performance of identified CSF metabolite biomarkers compared to the performance of OCGB status. Biomarkers are listed from highest to lowest AUC. Acc, Accuracy; AUC, area under the curve; CI, confidence interval; PPV, positive predictive value; ROC, receiver operator curve; NPV, negative predictive value; Sens, Sensitivity; Spec, specificity. Clinically defined converter *n* = 22, clinically defined non-converter *n* = 32.

To uncover further predictive biomarkers of conversion, NMR metabolomics analysis was used to simultaneously measure ∼50 small molecule, soluble metabolite levels in the baseline CSF samples. OPLS-DA revealed significant differences in the CSF metabolome of converters compared to non-converters (Fig. 2A), and external cross-validation confirmed that the accuracy of the multivariate metabolomics model (determined on independent test data) significantly outperformed the randomly permuted models (Supplementary Fig. 2). The metabolites driving the separation observed in the multivariate model included lactate and glucose, which were elevated in the converter CSF samples, and myo-inositol and creatine, which were decreased in converter CSF samples relative to non-converters (Fig. 2B–E). As the multivariate model, dominated by glucose, lactate, creatine and myo-inositol, was able to significantly predict conversion to CDMS (two-sided Kolmogorov–Smirnov test *P*-value < 0.001) we then investigated how each of these metabolites would perform in isolation, to determine if measuring a single biomarker could produce sufficient predictive accuracy for use in a clinical setting. Both myo-inositol and glucose CSF levels showed greater specificity for predicting the occurrence of a second clinical attack resulting in improved overall accuracy and AUC compared to OCGB status alone (Fig. 2F). By contrast, lactate and creatine did not perform better than OCGB status as predictors of conversion when measured in isolation. In addition, while lactate and creatine were important for discrimination in the multivariate model each of these metabolites in isolation did not reach significance by univariate analysis following correction for multiple comparisons.

### Multivariate proteomics analysis of baseline CSF samples identifies several proteins which outperform OCGB status, in terms of both sensitivity and specificity, for prediction of clinical conversion to MS

**Figure 3:**
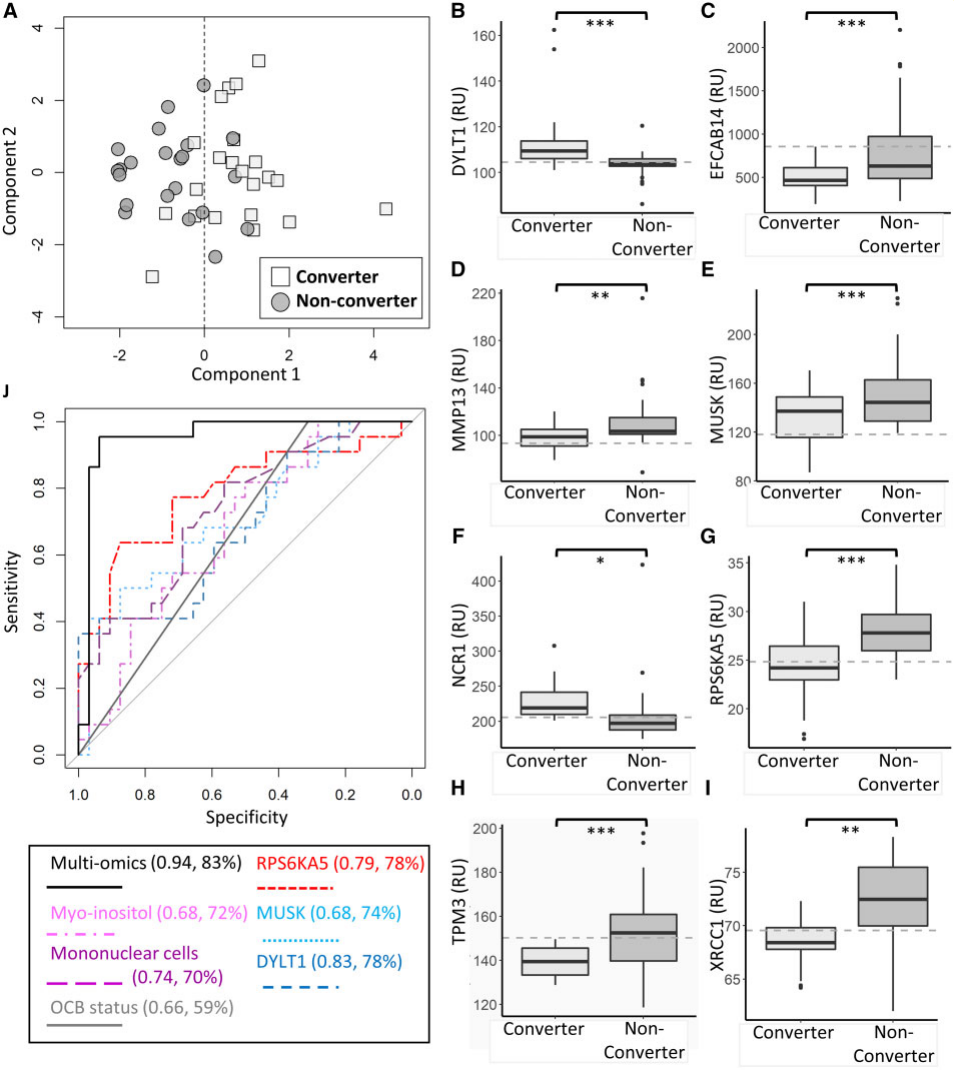
**Proteomics and multi-omics results.** (**A**) Representative OPLS-DA scores plot illustrating discrimination between clinically defined converters (square) and non-converter (circle) using CSF proteomics measurements. (**B–I**) Representative boxplots of the highest ranked significant discriminatory proteins identified by the OPLS-DA analysis in clinically defined converters and non-converters. Dashed lines represent the optimal threshold to achieve the greatest accuracy as determined by ROC analysis. For univariate analysis, two-sample *t*-tests were used for continuous variables while Chi-square tests were used for categorical variables as appropriate. While for multivariate analysis a two-sided Kolmogorov–Smirnov test was used to determine the significance of the OPLS-DA performance on independent test data relative to the null distribution. A Bonferroni correction to account for multiple comparisons was applied throughout. Univariate *P*-values below 0.05, 0.01 and 0.001 are represented by *, ** and ***, respectively. (**J**) ROC curves illustrating the performance of the multivariate model (solid black) compared to each component of the model alone. Protein markers RSK like protein kinase (dashed red), MUSK (dashed light blue), DYLT1 (dashed dark blue) along with myo-inositol (dashed pink) and CSF mononuclear levels (dashed purple) combined afford greater predictive accuracy than OCGB status (solid grey) alone. The AUC and accuracy of each ROC curve is displayed in brackets. Clinically defined converter *n* = 22, clinically defined non-converter *n* = 32.

The SomaScan^®^ platform was used to measure over 5000 CSF protein concentrations in both converters and non-converters. Multi-variate analysis confirmed significant separation between the converter and non-converter proteomes (Fig. 3A), which was validated by external cross-validation and permutation testing (Kolmogorov–Smirnov *P*-value < 0.001, Supplementary Fig. 3). The multi-variate analysis uncovered a panel of 89 proteins driving the discrimination between converter and non-converter in CIS CSF samples, 72 of which predict occurrence of a second attack with greater AUC (>0.66) than OCGB alone (Supplementary Table 1). Of the 89 protein biomarkers identified, 27 were elevated in converters at onset while the remaining 62 were elevated in the non-converter CSF samples relative to converters. Representative boxplots of the 12 most significant biomarkers are shown in Fig. 3B–I, while boxplots of the remaining discriminatory proteins can be found in Supplementary Fig. 5. ROC analysis was used to determine the optimum thresholds to produce the greatest predictive accuracy for each discriminatory protein identified.

DNA repair protein XRCC1 was significantly decreased in the converter CSF samples relative to non-converter (Fig. 3I) and was the highest performing biomarker overall; predicting conversion with an AUC, accuracy, sensitivity and specificity of 0.84, 80%, 73% and 84%, respectively. The top 20 most sensitive proteins are listed in Supplementary Table 2 while the top 20 most specific proteins are listed in Table 2. Both Tropomyosin α3-chain (TPM3) and EF-hand calcium-binding domain-containing protein 14 (EFCAB14) CSF levels were decreased in the converter cohort (Fig. 3H and C, respectively) and predicted conversion with 100% sensitivity (rivalling OCGB). Of note, TPM3 predicted conversion with a specificity, accuracy and AUC of 63%, 78% and 0.78, respectively, significantly outperforming OCGB. Eighty-eight of the identified protein biomarkers predict conversion with greater specificity than OCGB (top twenty illustrated in Table 2 full list Supplementary Table 1). In particular, muscle-skeletal receptor tyrosine-protein kinase (MUSK) levels were significantly decreased in converter samples relative to those of non-converters (Fig. 3E), and predicted conversion in this cohort with 100% specificity suggesting that this protein could be used in combination with OCGB to better predict the risk of a second clinical attack in CIS patients.

**Table 2:** Predictive performance of the top 20 identified CSF protein biomarkers with highest specificity compared to the performance of OCGB status.

	AUC [95% CI]	Acc	Sens	Spec	PPV	NPV	ROC threshold	Odds ratio	*P*-value
MUSK	0.68 [0.54–0.83]	0.74	0.36	1	1	0.7	118.1	36.57	0.02
MMP13	0.71 [0.57–0.86]	0.74	0.41	0.97	0.9	0.7	93.5	21.46	0.02
CCL17	0.69 [0.54–0.84]	0.76	0.5	0.94	0.85	0.73	106	15	0.03
CXCL1	0.72 [0.57–0.86]	0.72	0.41	0.94	0.82	0.7	1289.6	10.38	0.03
RARRES2	0.72 [0.58–0.86]	0.74	0.5	0.91	0.79	0.73	4102.7	9.67	0.01
SMDT1	0.72 [0.58–0.86]	0.74	0.5	0.91	0.79	0.73	23	9.67	0.02
THBS4	0.66 [0.51–0.81]	0.7	0.41	0.91	0.75	0.69	229.3	6.69	0.03
RPS6KA5	0.79 [0.66–0.92]	0.78	0.64	0.88	0.78	0.78	24.9	12.25	0.002
MFAP4	0.78 [0.65–0.91]	0.78	0.64	0.88	0.78	0.78	8757.5	12.25	0.003
IL22RA2	0.74 [0.6–0.88]	0.76	0.59	0.88	0.76	0.76	185.1	10.11	0.01
LCN10	0.74 [0.6–0.88]	0.74	0.55	0.88	0.75	0.74	58.7	8.4	0.004
SGCB	0.72 [0.58–0.86]	0.72	0.5	0.88	0.73	0.72	110.2	7	0.01
XRCC1	0.84 [0.72–0.95]	0.8	0.73	0.84	0.76	0.82	69.6	14.4	<0.001
MZF1	0.7 [0.55–0.85]	0.74	0.59	0.84	0.72	0.75	187.3	7.8	0.03
CCDC80	0.71 [0.56–0.85]	0.72	0.55	0.84	0.71	0.73	293.1	6.48	0.03
PLEKHA1	0.77 [0.63–0.9]	0.78	0.73	0.81	0.73	0.81	39.2	11.56	0.01
MZT1	0.74 [0.6–0.88]	0.76	0.68	0.81	0.71	0.79	68.8	9.29	0.01
TSSK2	0.74 [0.6–0.88]	0.72	0.59	0.81	0.68	0.74	79.8	6.26	0.005
PPIL2	0.72 [0.57–0.86]	0.72	0.59	0.81	0.68	0.74	94.1	6.26	0.03
COL6A2	0.71 [0.57–0.86]	0.72	0.59	0.81	0.68	0.74	28.8	6.26	0.01
OCGB status	0.66 [0.5–0.81]	59%	100%	31%	50%	100%	0.5	21	0.01

Proteins are listed from highest to lowest specificity.

Acc, Accuracy; AUC, area under the curve; CI, confidence interval; NPV, negative predictive value; PPV, positive predictive value; ROC, receiver operator curve; Sens, sensitivity; Spec, specificity.

### A linear combination of baseline CSF protein, metabolite and leukocyte concentrations predicts clinical conversion with an AUC of 0.94 and accuracy of 83%

As clinical chemistry, metabolomics and proteomics analysis of onset CSF samples revealed several predictive biomarkers of conversion which perform well individually, we next investigated whether a multivariate combination of the identified biomarkers and/or combining with OCGB provided improved predictive accuracy. Five variable multivariate models provided the greatest predictive accuracy (83%) with an AUC of 0.94; the addition of further variables provided no significant increase in accuracy. The variables selected by the multivariate model with greatest accuracy included MUSK, Ribosomal protein S6 kinase alpha-5 (RPS6KA5), Dynein light chain Tctex-type 1 (DYNLT1), CSF myo-inositol and mononuclear cell levels. While inclusion of mononuclear cell counts gave the highest performance, replacing this measure with leukocyte cell counts resulted in a decrease in accuracy of only 2%. The predictive accuracy of the multivariate multi-omics model greatly outperforms the accuracy of each identified biomarker in isolation (Fig. 3J). Interestingly, OCGB was not selected in the top performing model, likely due to the increased overall accuracy of baseline mononuclear and leukocyte cell counts in this cohort. Indeed, replacement of mononuclear cell count with OCGB in the multivariate model results in a decrease in predictive accuracy of 11%.

### Protein pathway enrichment analysis reveals perturbations in cytokine, TNF and leukocyte proliferation pathways in converters while proteins upregulated in non-converters are consistent with dysregulated cellular assembly and rheumatoid arthritis

**Figure 4:**
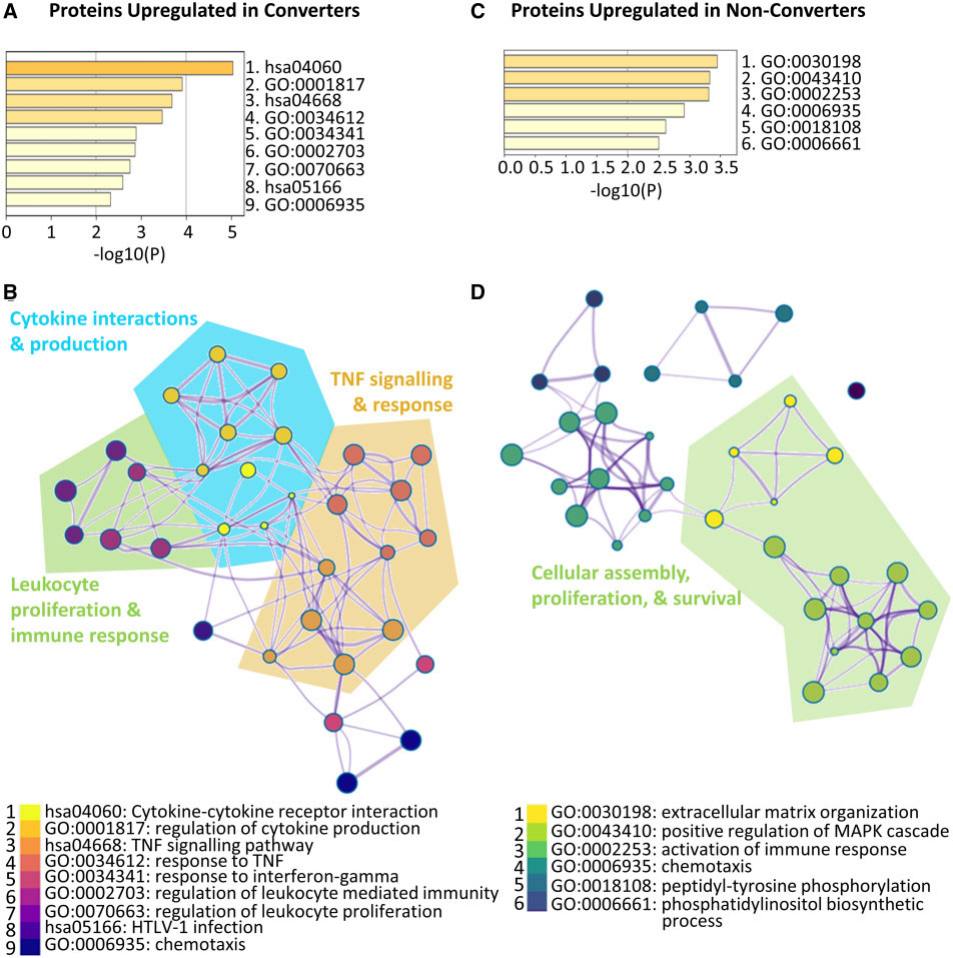
**Protein pathway enrichment analysis.** Top significant pathways associated with the identified proteins which are upregulated in clinically defined (**A)** converters and (**C)** non-converters. *P*-values <0.01 were considered significant. Visualization of the enrichment network associated with proteins upregulated in (**B)** converters (**D**) and non-converters. Nodes are coloured according to pathway while larger nodes represent smaller *P*-values. Similar and related pathways are grouped together. Clinically defined converter *n* = 22, clinically defined non-converter *n* = 32.

**Figure 5:**
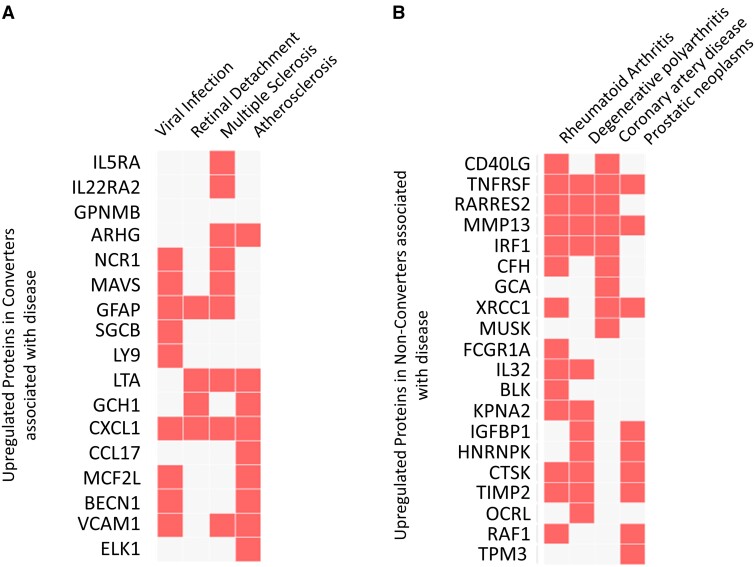
**Clustergram of associations of significantly enriched disease pathways with proteins upregulated in clinically defined** (**A**) converter CSF and (**B**) non-converter CSF. The proteins upregulated in converters were consistent with viral infection, retinal damage, and MS disease pathways while the proteins upregulated in non-converters suggest atypical rheumatoid arthritis presentation. Red squares represent a significant enrichment of the identified gene (rows) with a given pathological pathway (columns) while white squares represent no significant interaction. Fisher exact test, corrected *P*-values <0.05 were considered significant. Clinically defined converter *n* = 22, clinically defined non-converter *n* = 32.

Protein pathway enrichment analysis revealed that the top 89 discriminatory variables are linked through several pathways and physiological functions. The discriminatory proteins upregulated in the converter group relative to the non-converter group are consistent with perturbations in cytokine, TNF, and interferon-gamma signalling pathways along with leukocyte proliferation, leukocyte mediated immune response and chemotaxis (Fig. 4A). In contrast, those proteins elevated in the non-converters are consistent with cellular assembly, proliferation, and survival pathways including regulation of the MAPK cascade in addition to immune activation and chemotaxis pathways (Fig. 4B). These results point to distinct protein pathway perturbations in the converter and non-converter groups at baseline, suggesting potentially discrete underlying pathology. Of note, the proteins upregulated in the converters were enriched in disease pathways consistent with viral infection, retinal detachment, MS and atherosclerosis (Fig. 5A) suggesting that the proteome of the converter patients was representative of MS at onset. In contrast, the proteins upregulated in the non-converters are enriched in disease pathways consistent with rheumatoid arthritis, degenerative polyarthritis, coronary artery disease, and prostatic neoplasms (Fig. 5B).

## Discussion

This study provides a detailed exploration of predictive biomarkers in a prospective cohort of 54 individuals with CIS. Due to differences in past McDonald diagnostic criteria and the fact that the 2017 McDonald criteria have low specificity, the occurrence of a second attack was used to define clinical conversion in this cohort. This not only ensures that the predictive biomarkers identified are applicable to the ‘gold standard’ definition of MS, but also ensures clinical utility as those individuals who have a second clinical attack are more likely to develop severe permanent disability than those with ‘silent’ demyelinating events and, thus, should be treated as early as possible.

In total, only 41% of the patients recruited had a second attack and converted to clinically defined MS over the course of follow-up, which is somewhat lower than figures reported in a 20-year follow-up study where 63% of patients experienced a second clinical event.^3^ This is likely due to the maximum length of follow-up in this study, which was 10 years. The average time to conversion was 1.7 years and 72% of the converters had a second clinical attack within 2 years. As a result, only non-converters with a minimum of 2 years of follow-up were included in this study. No significant difference was observed in either the gender, age, or EDSS at onset between the converter and non-converter groups.

Previous reports suggest that between 61 and 68.9% of patients with CIS test positive for OCGB^49^ and 50% of these have a second clinical attack within 4 years of onset.^16^ In line with this, 81% of the CIS patients in our cohort tested positive for OCGB at baseline, of which, 50% converted to clinically defined MS. In contrast, none of the patients in the converter group tested negative for OCGB at baseline. As a result, the use of OCGB in isolation predicted occurrence of a second clinical attack with 100% sensitivity but only 31% specificity resulting in an overall accuracy and AUC of 59% and 0.66, respectively.

CSF leukocyte cell count was moderately elevated (>4 but < 30 cells/µl) in 54% of the cohort at baseline. A larger proportion of the patients who went on to have a second clinical attack (72%) exhibited elevated leukocyte cell counts at baseline when compared to those who did not clinically convert over the follow up period (41%). As a result, a significant elevation in leukocyte cell counts was observed in converter CSF samples relative to non-converters, which is consistent with an increased immune response at baseline in these individuals. Interestingly, when the total leukocyte population was evaluated at the cell-type level, a significant elevation in the mononuclear, but not PMN cells, was observed in the converter group suggesting that the elevated leukocyte levels are dominated by changes in mononuclear cells. Leukocytes are known to be moderately elevated (4–50 cells/mm^3^) in up to 60% of MS cases^50^ while relapsing remitting (RR) MS patients with pleocytic CSF (>5 cells/mm^3^) are known to have increased annualized relapse rates.^51^ Indeed, using leukocyte levels >4 cells/mm^3^ predicted transition to clinically defined MS with an accuracy of 65%, although ROC analysis revealed that a threshold of 3.5 cells/mm^3^ was optimal (Fig. 1G). Interestingly, the mononuclear cell population had greater predictive value than leukocyte cell count. While less sensitive, both CSF mononuclear and leukocyte levels predicted the occurrence of second clinical attack with higher accuracy than OCGB (70% and 67%, respectively). As these parameters are routinely measured in order to rule out other potential diagnoses, this suggests that inclusion of mononuclear cells as an adjunct to MRI parameters and OCGB could improve identification of those at high risk of clinically converting to clinically defined MS without the requirement for an additional biochemical test.

Changes in the concentration of soluble, small molecule metabolites in biofluid samples, including CSF, are known to be associated with CNS pathology, increased inflammation, and an elevated immune response. We and others have demonstrated that relatively subtle differences in the pathological mechanisms of inflammatory and demyelinating CNS disease are detectable in the peripheral metabolome.^35^^,^^52^^,^^53^ Multivariate analysis revealed that myoinositol, glucose, creatine and lactate levels discriminate between converters and non-converters. Converters had significantly higher average CSF myo-inositol and glucose levels. While lactate and creatine did not reach univariate significance when corrected for multiple comparisons the average lactate levels were higher and creatine levels lower in converters compared to non-converters at baseline. Perturbations in glucose and lactate CSF levels in the converter group are consistent with dysregulated CNS energy metabolism. Perturbed energy metabolism in MS patients has been previously reported^54^ and linked to oxidative damage, mitochondrial function imbalance, and neuroaxonal degeneration. Increased CSF lactate levels have been observed in RRMS patients and correlate with markers of neuroaxonal damage.^55^ Under healthy conditions, the brain utilizes 25% of the body’s total glucose^56^ which is converted to lactate by anaerobic glycolysis within glial cells and shuttled to neurons as the primary energy source. Ineffective nerve conductance as a result of demyelination coupled with CNS inflammation in MS results in an increased neuronal energy demand.^57^ Thus, the metabolite changes observed may represent increased neuronal energy metabolism dysfunction in those who go on to have a second clinical attack.

Myo-inositol is a component of plasma membranes and myelin^58^ and thus the increased levels of myo-inositol observed in the converter cohort may be a direct result of myelin breakdown in the CNS. In addition, an increased level of myo-inositol is known to be a marker of gliosis^59^ and elevated levels of myo-inositol have been observed in MS and CIS lesions.^60^ This could suggest that CIS patients, who go on to have a second clinical attack, have greater demyelination and glial activation at baseline relative to non-converter which, while detectable biochemically, is not yet distinguishable radiologically or clinically.

Over 5000 proteins were measured in the baseline CSF samples using the SomaScan^®^ platform to uncover novel predictive biomarkers of clinically defined conversion. Multivariate pattern recognition identified 89 protein biomarkers. Protein pathway enrichment analysis confirmed that the proteins upregulated in the clinically defined converter group were consistent with increased inflammation and an altered immune response. Significant enrichment in pathways regulating leukocyte proliferation and immunity is consistent with the elevated white blood cell counts and increased gliosis observed in these patients. Furthermore, the protein biomarkers identified were significantly enriched in regulation of cytokine production and receptor signalling, TNF signalling, and response to interferon gamma. Investigation of disease associated protein pathways revealed that the proteins elevated in the converter CSF were significantly enriched in MS suggesting that the proteins identified are indeed able to identify MS at the point of first attack. In addition, viral infection pathways were significantly enriched in the converter cohort which may correspond to virus-associated onset that is observed in many MS cases. In contrast, the proteins upregulated in the non-converter cohort were significantly enriched in pathways associated with cellular assembly, proliferation, and survival. Pathways associated with activation of the immune response were also significantly enriched in the non-converters at baseline. Interestingly, disease pathways associated with the proteins elevated in the non-converter group were associated with rheumatoid arthritis and degenerative arthritis as well as coronary artery disease, suggesting some individuals in this group may have atypical presentation of peripheral inflammatory, immune-mediated disease. Indeed, recent evidence suggests that chronic peripheral inflammation can lead to blood brain barrier dysfunction and CNS involvement in diseases such as rheumatoid arthritis.^61^

The proteins with greatest overall predictive power were DNA repair protein XRCC1 (XRCC1), dynein light chain Tctex-TYPE 1 (DYNLT1), and natural cytotoxicity triggering receptor 1 (NCR1) with AUC values of 0.84, 0.84 and 0.83, respectively. Partial loss of XRCC1 renders brain cells vulnerable to oxidative damage^62^ suggesting that the decreased levels of XRCC1 in the converters could reflect an increased propensity to CNS oxidative damage in this cohort. This is consistent with oxidative damage and demyelinating induced perturbations in energy metabolism observed in the metabolomics analysis. DYNLT1 regulates neuronal morphogenesis and, during cortical development, inhibits neurogenesis.^63^ Thus, increased levels of DYNLT1 in converter CSF may reflect increased inhibition of neurogenesis in this group or dysregulation of neuronal architecture. Increased levels of NCR1 in the converter group may represent increased activation of natural killer (NK) cells.^64^ The majority of NCR1 expression in the CNS is localized to astrocytes^65^ supporting the metabolomics results which were suggestive of increased gliosis in the converter cohort. While NK cell activation in the CNS had been implicated in several autoimmune diseases^66^ their role in MS remains to be elucidated.

The extensive biomarker discovery employed here successfully identified clinical chemistry, metabolite, and protein biomarkers of conversion to clinically defined MS, each of which perform well in isolation and provide novel insight into the different pathological mechanisms in converters and non-converters at baseline. Future work, on larger prospective cohorts, will investigate whether the biomarkers identified here could be included in the McDonald criteria to improve specificity when identifying patients at high risk of second clinical attack. In particular, the data presented suggest that a larger follow-on study investigating the impact of leukocyte and mononuclear cell counts on prediction of clinical conversion would be particularly attractive as these measures are routinely available. Ongoing work will validate the identified biomarkers in the further independent cohorts, develop a method to measure the identified biomarkers using a single, cost-effective, assay for use in a clinical setting, and compare this test with other recently identified predictive biomarkers including baseline MRI findings, clinical variables, NfL and IgG/M levels.

## Supplementary material

Supplementary material is available at *Brain Communications* online.

## Funding

F.P. received funding from the Multiple Sclerosis Society UK (grant 59) and the Medical Research Council (MC_PC_15029). T.Y. is supported by the Ministry of Health, Singapore through the National Medical Research Council Research Training Fellowship (NMRC/Fellowship/0038/2016).

## Competing interests

Y.Z., M.S., S.A., T.D.W.C., R.H., J.O., D.L. and D.C.A. declare no competing interests. S.A. declares no competing interests. J.K. received speaker fees, research support, travel support, and/or served on advisory boards by ECTRIMS, Swiss MS Society, Swiss National Research Foundation, (320030_160221), University of Basel, Bayer, Biogen, Celgene, Merck, Novartis, Roche, Sanofi. FP has received travel awards from ECTRIMS, Merck, and the Multiple Sclerosis Society UK. T.Y. has received travel grants from UCB, Merck and PACTRIMS, and travel awards from ECTRIMS, ACTRIMS and Orebro University. J.P. is partly funded by highly specialized services to run a national congenital myasthenia service and a neuromyelitis service. She has received support for scientific meetings and honorariums for advisory work from Merck Serono, Biogen Idec, Novartis, Teva, Chugai Pharma and Bayer Schering, Alexion, Roche, Genzyme, MedImmune, EuroImmun, MedDay, Abide ARGENX, UCB and Viela Bio and grants from Merck Serono, Novartis, Biogen Idec, Teva, Abide, MedImmune, Bayer Schering, Genzyme, Chugai and Alexion. She has received grants from the MS society, Guthrie Jackson Foundation, NIHR, Oxford Health Services Research Committee, EDEN, MRC, GMSI, John Fell and Myaware for research studies.

## Supplementary Material

fcab084_Supplementary_Data

## Abbreviations

AUC =: area under the curve
CIS =: clinically isolated syndrome
EDSS =: expanded disability scale status
IQR =: interquartile range
MS =: multiple sclerosis
NK =: natural killer
NMR =: nuclear magnetic resonance
OCGB =: oligoclonal bands
OPLS-DA =: orthogonal partial least squares discriminant analysis
PIRA =: disability progression independent of relapse activity
PMN =: polymorphonuclear
Q_alb_ =: CSF/serum albumin ratio
ROC =: receiver operator curves
RR =: relapsing remitting
VIP =: variable importance projection score
