# Supplementary material for: Integrative biochemical, proteomics and metabolomics cerebrospinal fluid biomarkers predict clinical conversion to multiple sclerosis
Source: Brain Commun. 2021 Apr 19;3(2):fcab084. doi: 10.1093/braincomms/fcab084 (PMC8111065; doi:10.1093/braincomms/fcab084)
Supplement: fcab084_Supplementary_Data [file fcab084_supplementary_data.pdf]

Supplementary Information.

Integrative biochemical, proteomics, and metabolomics cerebrospinal fluid biomarkers predict clinical conversion to multiple sclerosis.

Running head: Integrative biomarkers predict conversion to MS

Fay Probert PhD[1,5], Tianrong Yeo MD, PhD[1,2], Yifan Zhou MSc[1], Megan Sealey PhD[1], Siddharth Arora PhD[3], Jacqueline Palace MD[4], Timothy DW Claridge PhD[5], Rainer Hillenbrand PhD[6], Johanna Oechtering MD[7], David Leppert MD[7], Jens Kuhle MD, PhD[7], Daniel C Anthony PhD[1].

Affiliations

1 Department of Pharmacology, University of Oxford, UK.
2 Department of Neurology, National Neuroscience Institute, Singapore.
3 Mathematical Institute, University of Oxford, UK.
4 Nuffield Department of Clinical Neurosciences, John Radcliffe Hospital, University of Oxford, UK.
5 Department of Chemistry, University of Oxford, UK.
6 Novartis Pharma AG, Basel, Switzerland.
7 Neurology, Departments of Medicine, Clinical Research and Biomedicine, University Hospital Basel, University of Basel, Switzerland.

Correspondence to: Jens Kuhle (Jens.Kuhle@usb.ch).

Correspondence may also be sent to: Daniel Anthony (Daniel.anthony@pharm.ox.ac.uk)

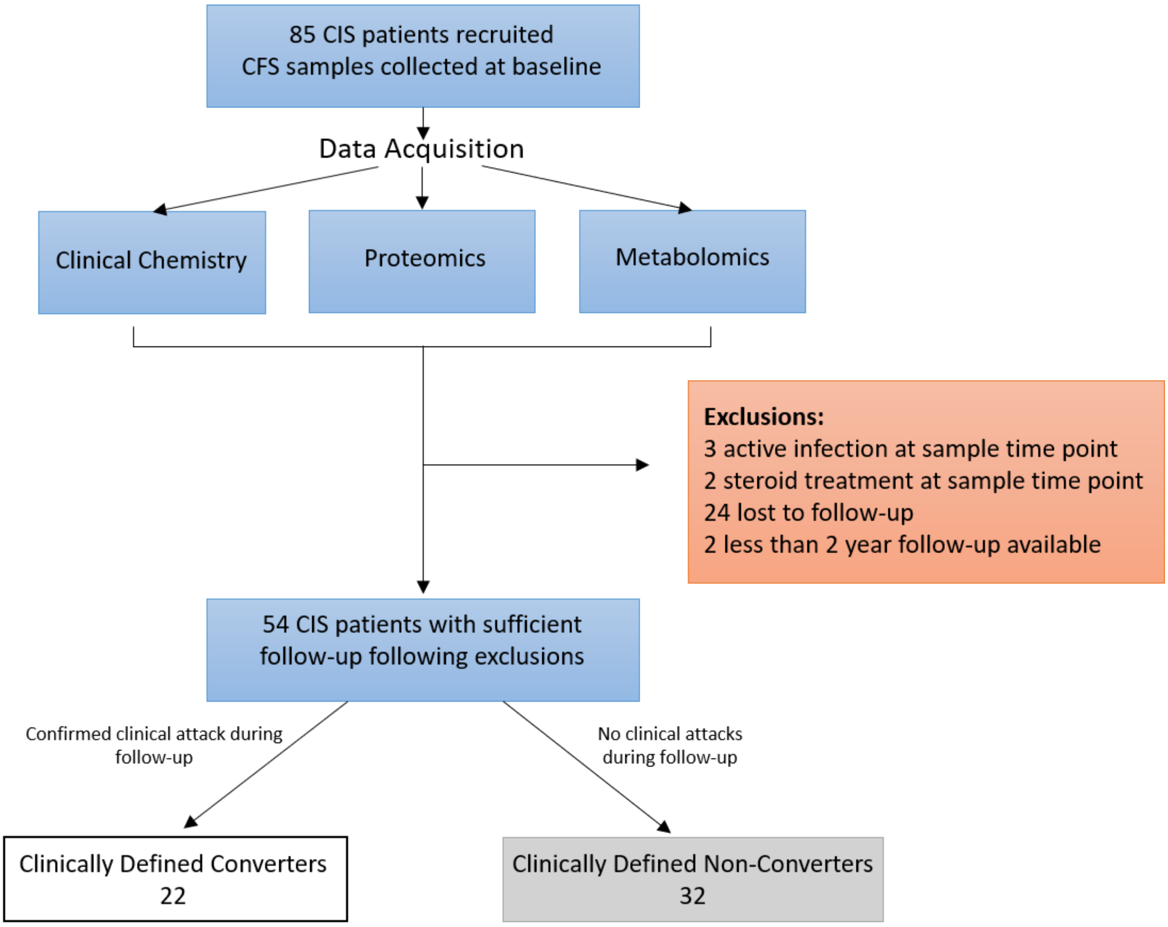

Supplementary Figure 1. Flow diagram illustrating numbers of individuals included in the study and reasons for exclusion. 85 patients were recruited at CIS onset, CSF samples were collected at baseline and clinical chemistry, proteomics, and metabolomics data was acquired on all samples. 26 of the patients either had no follow-up data or less than 2 years of follow-up and thus were excluded from the analysis. Three patients were confirmed to have active infections and two were receiving steroid treatment at the time of CSF sampling and were excluded from the analysis. Thus, a total of 54 patient samples were confirmed to be eligible analysis and were stratified into clinically defined converter and non-converter groups using the follow-up data.

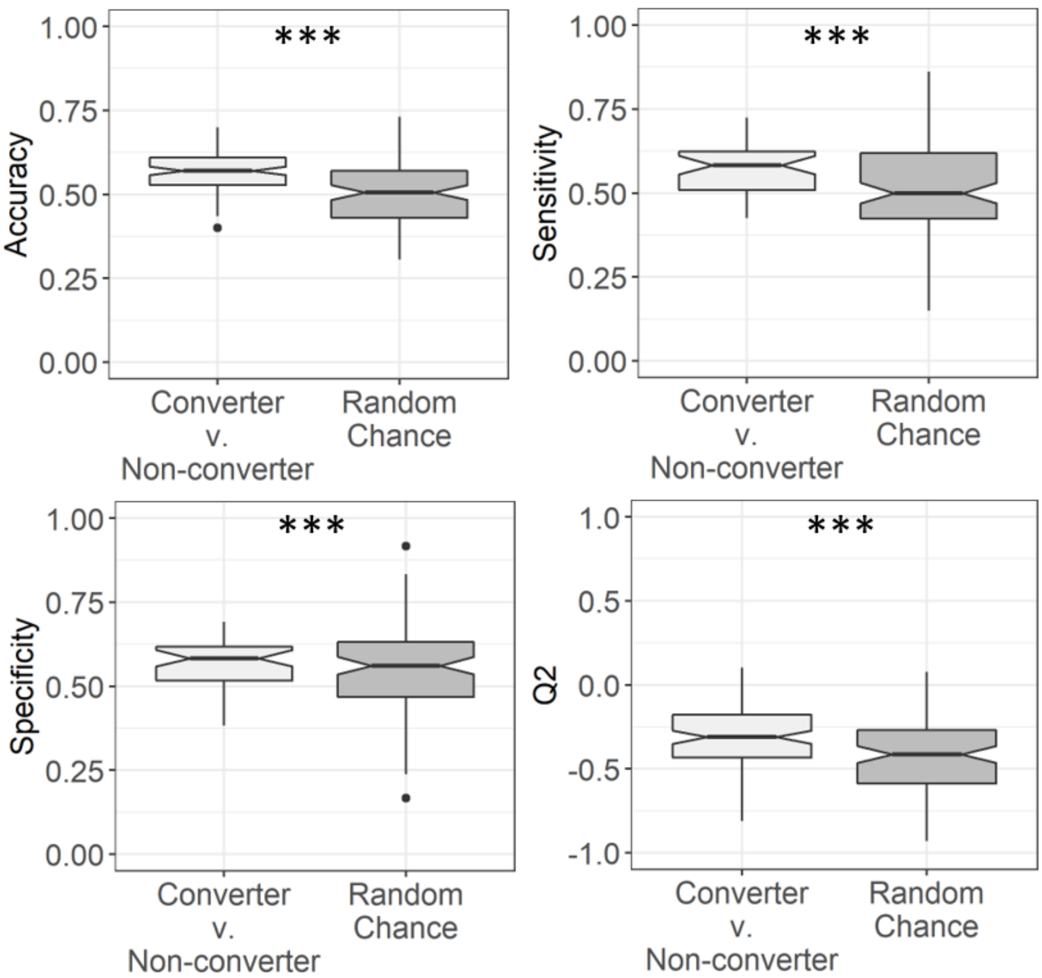

Supplementary Figure 2. Validating metabolomics OPLS-DA models on independent test data. 10-fold cross-validation with repetition reveals significantly increased accuracy, sensitivity, specificity and Q2 on independent test data (excluded when training the model) relative to the null distribution produced by permutation testing (the performance expected by random chance alone). Two-sample Kolmogorov-Smirnov p-values < 0.001 are represented by ***.

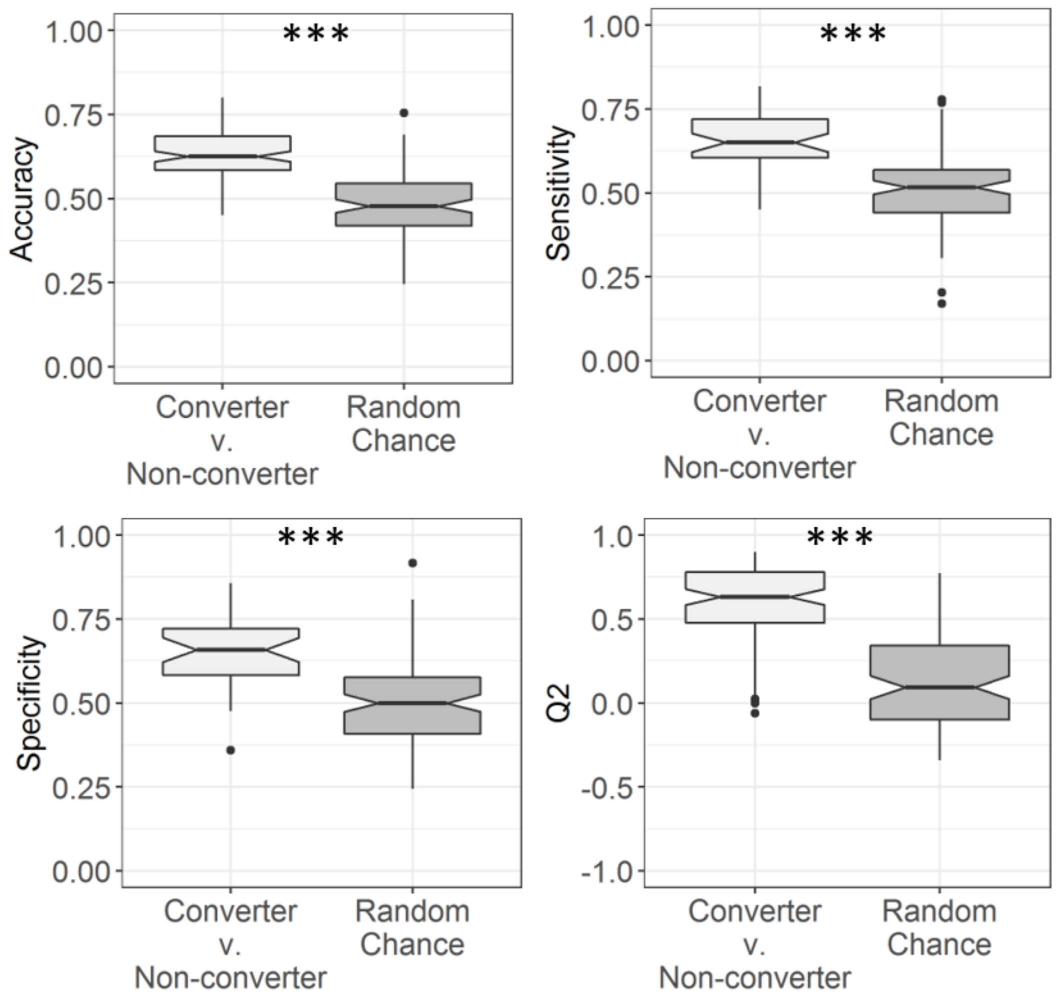

Supplementary Figure 3. Validating proteomics OPLS-DA models on independent test data. 10-fold cross-validation with repetition reveals significantly increased accuracy, sensitivity, specificity and Q2 on independent test data (excluded when training the model) relative to the null distribution produced by permutation testing (the performance expected by random chance alone). Two-sample Kolmogorov-Smirnov p-values < 0.001 are represented by ***.

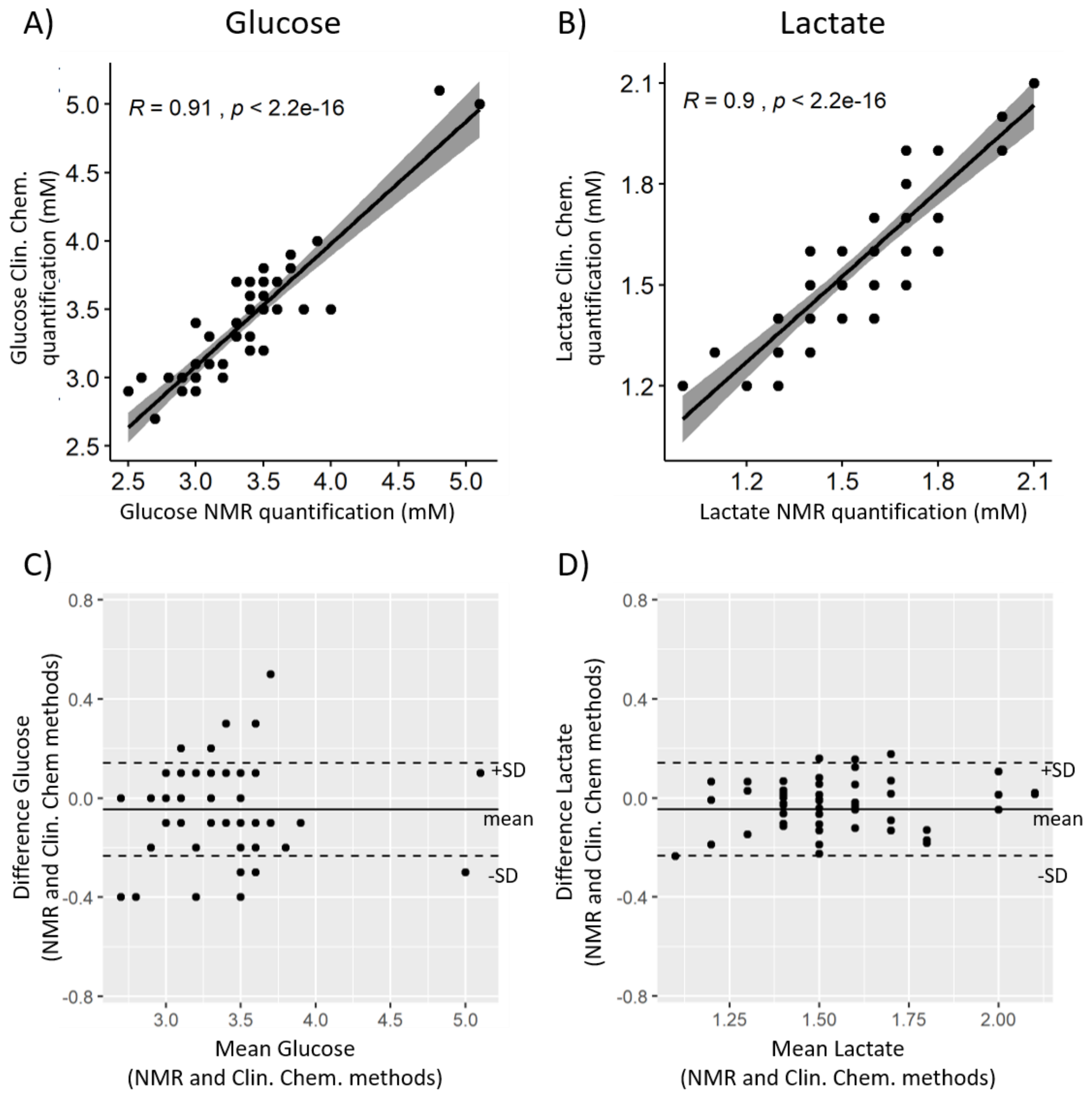

Supplementary Figure 4. Agreement between metabolite concentrations determined by NMR metabolomics and standard laboratory chemistry methods. Significant Pearson linear correlations were observed for (A) glucose and (B) lactate concentrations. (C-D) Bland-Altman plots illustrate excellent agreement between the two measurement methods.

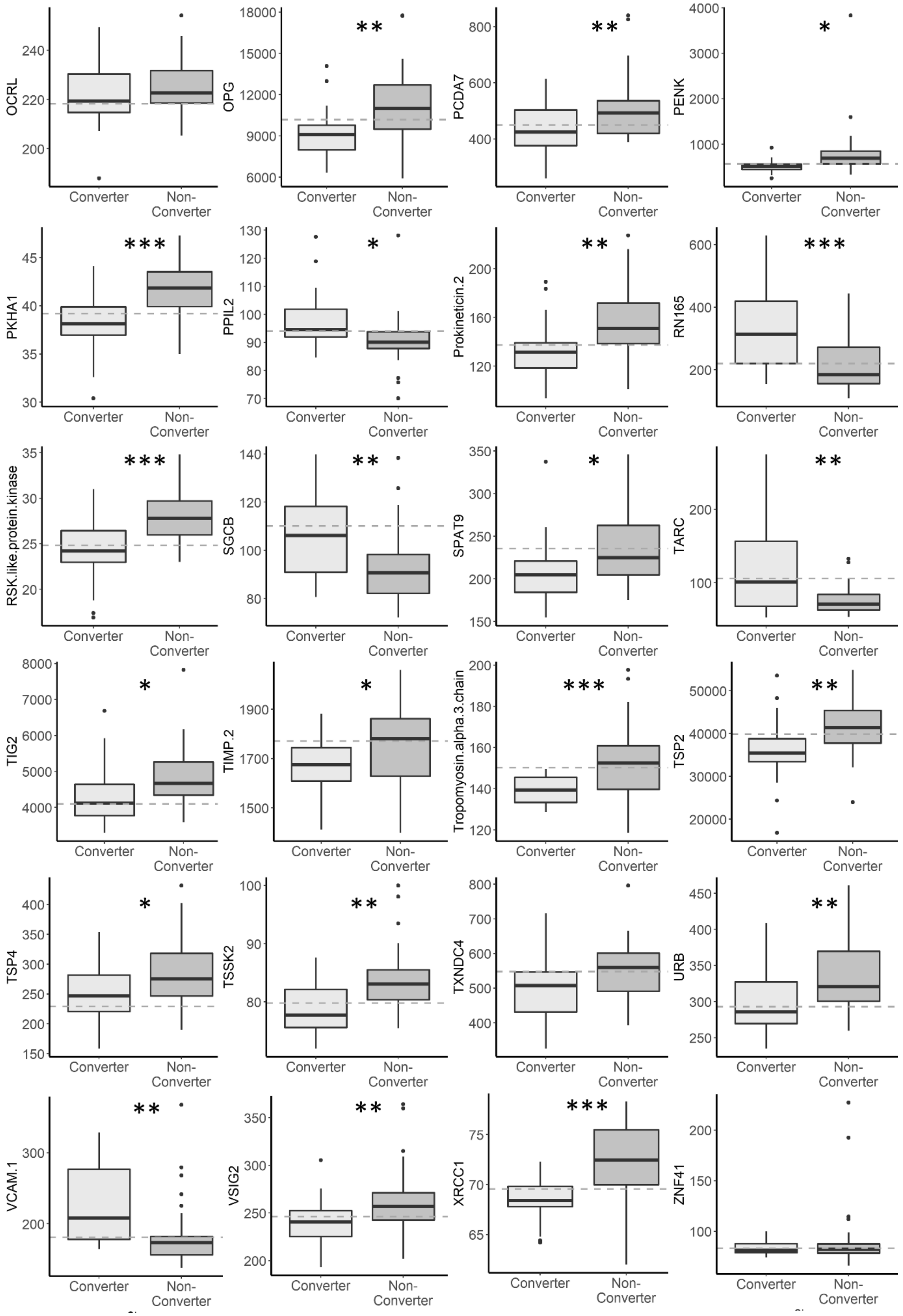

OCRL
Converter
Non-Converter
OPG
**
PCDA7
**
PENK
*
PKHA1
***
PPIL2
*
Prokineticin.2
**
RN165
***
RSK.like.protein.kinase
***
SGCB
**
SPAT9
*
TARC
**
TIG2
*
TIMP.2
*
Tropomyosin.alpha.3.chain
***
TSP2
**
TSP4
*
TSSK2
**
TXNDC4
URB
**
VCAM.1
**
VSIG2
**
XRCC1
***
ZNF41

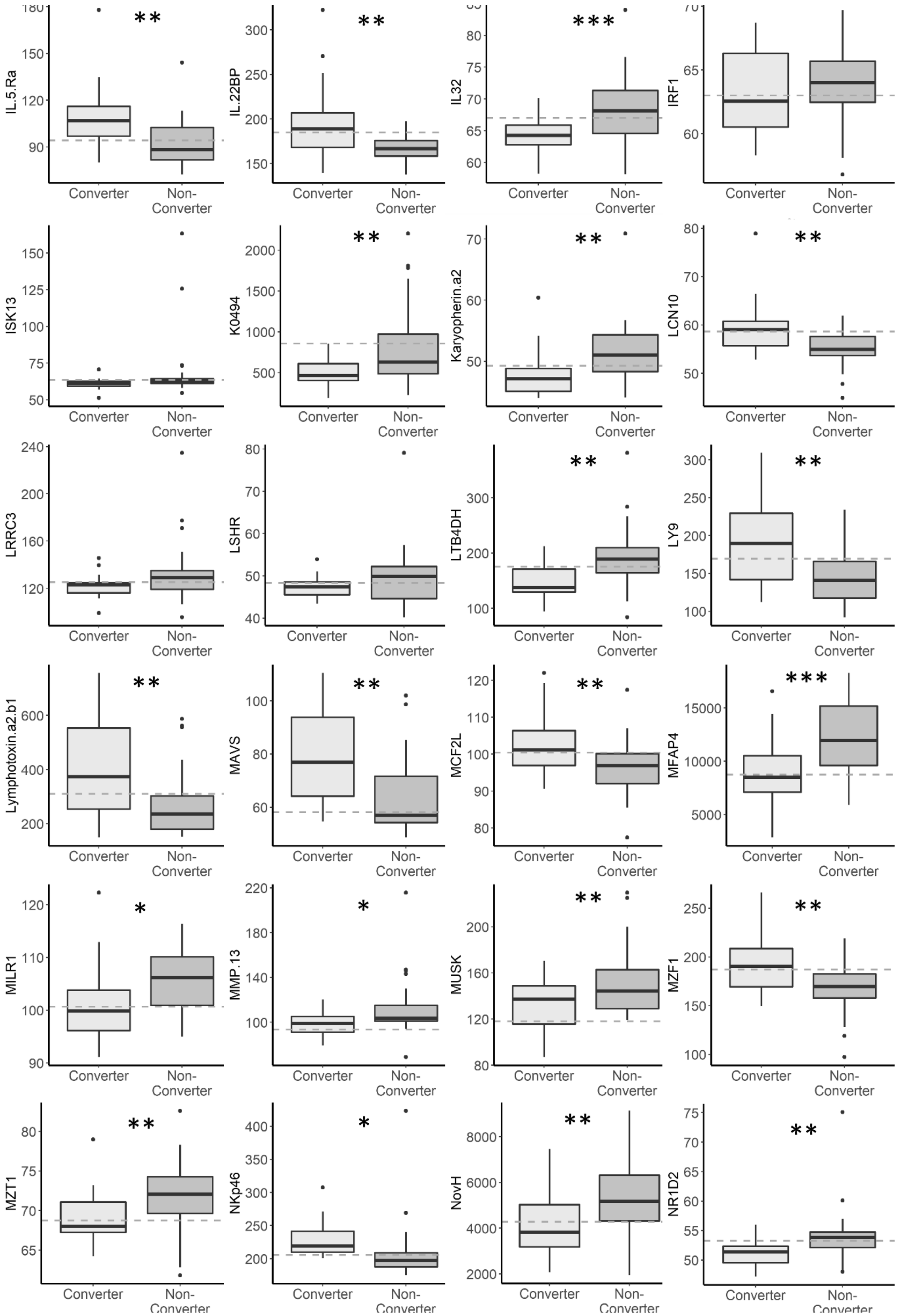
IL.5.Ra
**
IL.22BP
**
IL32
***
IRF1
ISK13
K0494
**
Karyopherin.a2
**
LCN10
**
LRRC3
LSHR
LTB4DH
**
LY9
**
Lymphotoxin.a2.b1
**
MAVS
**
MCF2L
**
MFAP4
***
MILR1
*
MMP.13
*
MUSK
**
MZF1
**
MZT1
**
NKp46
*
NovH
**
NR1D2
**
Converter
Non-Converter

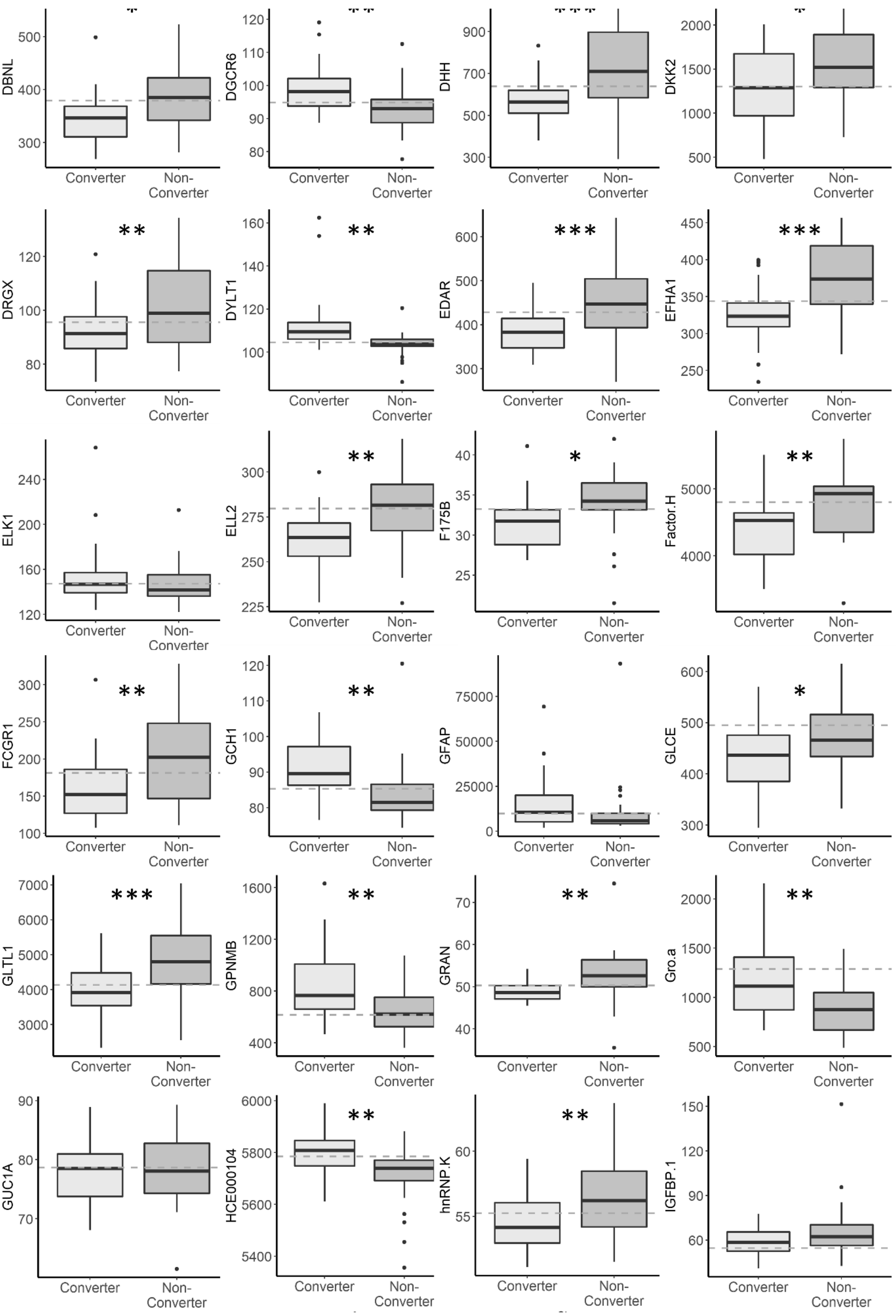

DBNL
DGCR6
**
DHH
DKK2
DRGX
**
DYLT1
**
EDAR
***
EFHA1
***
ELK1
ELL2
**
F175B
*
Factor.H
**
FCGR1
**
GCH1
**
GFAP
GLCE
*
GLTL1
***
GPNMB
**
GRAN
**
Gro.a
**
GUC1A
HCE000104
**
hnRNP.K
**
IGFBP.1
Converter
Non-Converter

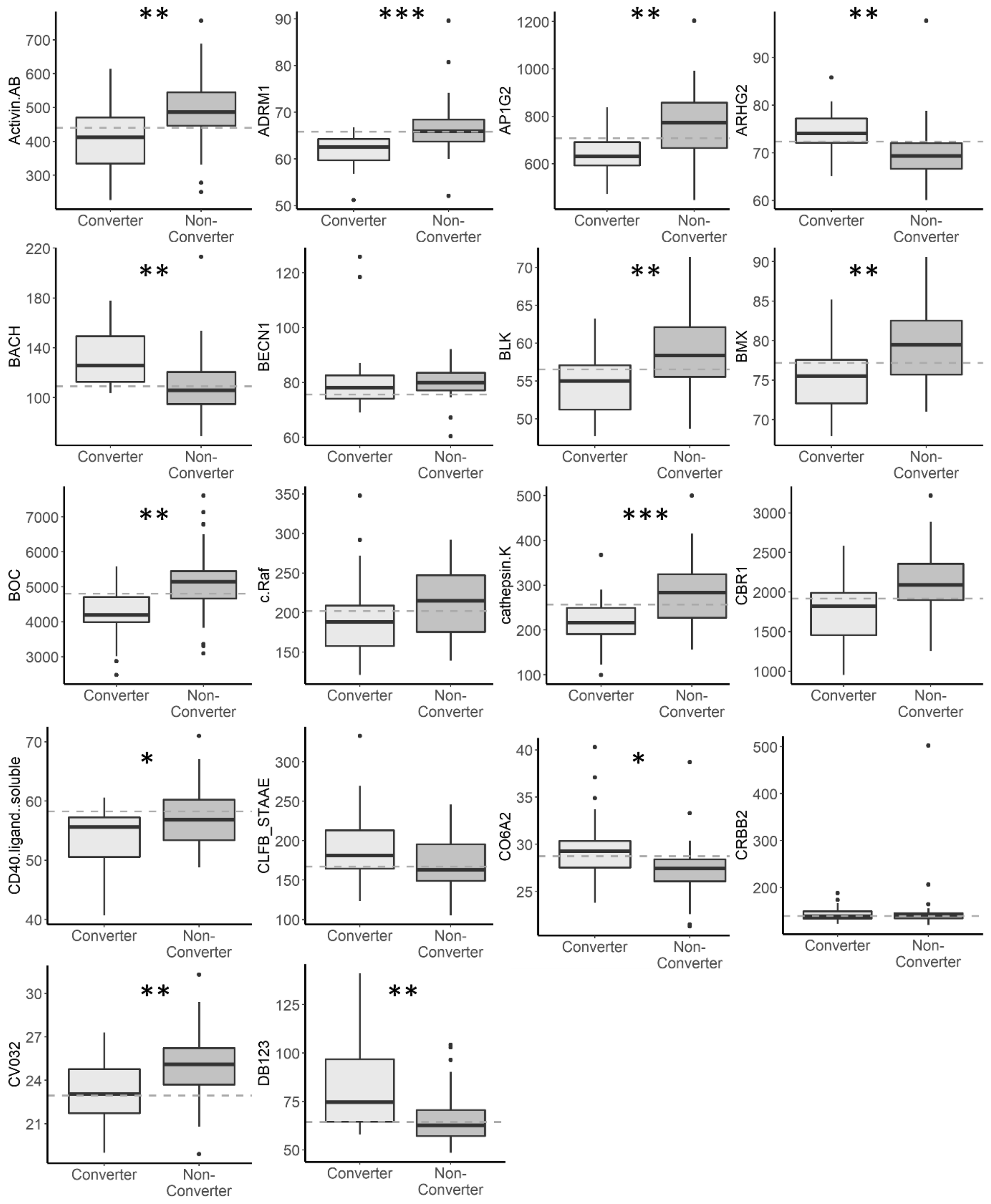

Supplementary Figure 5. Boxplots of the 89 significant discriminatory proteins identified by the OPLS-DA analysis in converters (white) and non-converters (grey). Univariate p-values below 0.05, 0.01, and 0.001 are represented by *, **, *** respectively.

Supplementary Table 1. Predictive performance of 89 identified CSF protein biomarkers identified by multivariate analysis. Biomarkers are listed from highest to lowest AUC. Abbreviations: AUC, area under the curve; CI, confidence interval, Acc, Accuracy; Sens, Sensitivity; Spec, specificity; PPV, positive predictive value; NPV, negative predictive value; ROC, receiver operator curve.

Uniprot #	Protein	Gene ID	Perturbation	AUC [95% CI]	Acc	Sens	Spec	PPV	NPV	ROC threshold	Odds Ratio	P-value
**P63172**	Dynein light chain Tctex-type 1	DYNLT1	upregulated in converter	0.83 [0.71-0.95]	78%	92%	67%	69%	91%	104.5	22.00	<0.001
**P18887**	DNA repair protein XRCC1	XRCC1	upregulated in non-converter	0.84 [0.72-0.95]	80%	82%	76%	84%	73%	69.6	14.40	<0.001
**O76036**	Natural cytotoxicity triggering receptor 1	NCR1	upregulated in converter	0.79 [0.66-0.92]	80%	92%	69%	72%	91%	205.5	25.56	<0.001
**P01210**	Proenkephalin-A	PENK	upregulated in non-converter	0.78 [0.65-0.91]	78%	83%	71%	78%	77%	570.2	12.14	0.001
**P28676**	Grancalcin	GCA	upregulated in non-converter	0.77 [0.63-0.9]	74%	82%	65%	72%	77%	50.4	8.69	0.001
**Q6ZSG1**	E3 ubiquitin-protein ligase RNF165	RNF165	upregulated in converter	0.84 [0.72-0.95]	72%	81%	63%	69%	77%	219.7	7.48	0.002
**O75582**	Ribosomal protein S6 kinase alpha-5	RPS6KA5	upregulated in non-converter	0.79 [0.67-0.92]	78%	78%	78%	88%	64%	24.9	12.25	0.002
**P06753**	Tropomyosin alpha-3 chain	TPM3	upregulated in non-converter	0.69 [0.54-0.84]	78%	100%	65%	63%	100%	150.3	73.33	0.001

**Q16186**	Proteasomal ubiquitin receptor ADRM1	ADRM1	upregulated in non-converter	0.74 [0.6-0.88]	72%	90%	61%	59%	91%	65.9	14.62	0.003
**Q7Z434**	Mitochondrial antiviral-signalling protein	MAVS	upregulated in converter	0.73 [0.59-0.87]	70%	90%	59%	56%	91%	58.2	12.86	0.002
**P30793**	GTP cyclohydrolase 1	GCH1	upregulated in converter	0.74 [0.6-0.88]	76%	85%	67%	72%	82%	85.3	11.50	0.003
**Q92974**	Rho guanine nucleotide exchange factor 2	ARHGEF2	upregulated in converter	0.74 [0.6-0.88]	76%	81%	70%	78%	73%	72.4	9.52	0.002
**P52292**	Importin subunit alpha-1	KPNA2	upregulated in non-converter	0.78 [0.65-0.91]	72%	81%	63%	69%	77%	49.3	7.48	0.003
**P55083**	Microfibril-associated glycoprotein 4	MFAP4	upregulated in non-converter	0.78 [0.65-0.91]	78%	78%	78%	88%	64%	8757.5	12.25	0.003
**O00154**	Cytosolic acyl coenzyme A thioester hydrolase	ACOT7	upregulated in converter	0.78 [0.65-0.91]	74%	91%	63%	63%	91%	109.2	16.67	0.002
**Q14995**	Nuclear receptor subfamily 1 group D member 2	NR1D2	upregulated in non-converter	0.68 [0.54-0.83]	72%	90%	61%	59%	91%	53.3	14.62	0.004
**Q9HB21**	Pleckstrin homology domain-containing family A member 1	PLEKHA1	upregulated in non-converter	0.71 [0.57-0.86]	78%	81%	73%	81%	73%	39.2	11.56	0.01
**P19320**	Vascular cell adhesion protein 1	VCAM1	upregulated in converter	0.72 [0.58-0.86]	72%	79%	64%	72%	73%	181.1	6.81	0.001
**Q9UNE0**	Tumor necrosis factor receptor superfamily member EDAR	EDAR	upregulated in non-converter	0.72 [0.58-0.86]	72%	84%	62%	66%	82%	428.6	8.59	0.003

**P43235**	Cathepsin K	CTSK	upregulated in non-converter	0.74 [0.6-0.88]	72%	84%	62%	66%	82%	257.1	8.59	0.01
**Q8IYU8**	Calcium uptake protein 2, mitochondrial	MICU2	upregulated in non-converter	0.7 [0.55-0.85]	74%	82%	65%	72%	77%	344.1	8.69	0.005
**P24001**	Interleukin-32	IL32	upregulated in non-converter	0.72 [0.57-0.86]	72%	90%	61%	59%	91%	67	14.62	0.004
**Q14914**	Prostaglandin reductase 1	PTGR1	upregulated in non-converter	0.75 [0.61-0.89]	74%	85%	64%	69%	82%	175.5	9.90	0.002
**O75843**	AP-1 complex subunit gamma-like 2	AP1G2	upregulated in non-converter	0.75 [0.62-0.89]	74%	85%	64%	69%	82%	708.5	9.90	0.01
**Q01344**	Interleukin-5 receptor subunit alpha	IL5RA	upregulated in converter	0.75 [0.61-0.89]	74%	82%	65%	72%	77%	94.3	8.69	0.01
**Q8N688**	Beta-defensin 123	DEFB123	upregulated in converter	0.79 [0.67-0.92]	69%	80%	59%	63%	77%	64.4	5.67	0.01
**Q8N428**	Polypeptide N-acetylgalactosaminyltransferase 16	GALNT16	upregulated in non-converter	0.75 [0.62-0.89]	74%	78%	68%	78%	68%	4138.8	7.65	0.005
**P01374**	Lymphotoxin-alpha	LTA	upregulated in converter	0.76 [0.62-0.89]	74%	78%	68%	78%	68%	310.4	7.65	0.005
**Q9BWV1**	Brother of CDO	BOC	upregulated in non-converter	0.72 [0.57-0.86]	74%	85%	64%	69%	82%	4808.5	9.90	0.01
**P35442**	Thrombospondin-2	THBS2	upregulated in non-converter	0.71 [0.56-0.85]	72%	84%	62%	66%	82%	39830.9	8.59	0.004

**Q9HC23**	Prokineticin-2	PROK2	upregulated in non-converter	0.73 [0.59-0.87]	76%	81%	70%	78%	73%	137.4	9.52	0.005
**Q08AG7**	Mitotic-spindle organizing protein 1	MZT1	upregulated in non-converter	0.74 [0.6-0.88]	76%	79%	71%	81%	68%	68.8	9.29	0.01
**P51813**	Cytoplasmic tyrosine-protein kinase BMX	BMX	upregulated in non-converter	0.74 [0.6-0.88]	69%	78%	59%	66%	73%	77.2	5.09	0.02
**Q969J5**	Interleukin-22 receptor subunit alpha-2	IL22RA2	upregulated in converter	0.75 [0.61-0.88]	76%	76%	76%	88%	59%	185.1	10.11	0.01
**Q6JVE6**	Epididymal-specific lipocalin-10	LCN10	upregulated in converter	0.77 [0.64-0.91]	74%	74%	75%	88%	55%	58.7	8.40	0.004
**Q96PF2**	Testis-specific serine	TSSK2	upregulated in non-converter	0.72 [0.58-0.86]	72%	74%	68%	81%	59%	79.8	6.26	0.005
**P08603**	Complement factor H	CFH	upregulated in non-converter	0.78 [0.65-0.91]	72%	90%	61%	59%	91%	4803.9	14.62	0.01
**O00472**	RNA polymerase II elongation factor ELL2	ELL2	upregulated in non-converter	0.79 [0.66-0.92]	69%	89%	57%	53%	91%	279.8	11.33	0.01
**O00300**	Tumor necrosis factor receptor superfamily member 11B	TNFRSF11B	upregulated in non-converter	0.71 [0.57-0.86]	74%	85%	64%	69%	82%	10188.4	9.90	0.01
**O43323**	Desert hedgehog protein	DHH	upregulated in non-converter	0.72 [0.57-0.86]	72%	84%	62%	66%	82%	639	8.59	0.01
**Q14956**	Transmembrane glycoprotein NMB	GPNMB	upregulated in converter	0.76 [0.62-0.9]	65%	84%	54%	50%	86%	616.9	6.33	0.02

**Q15018**	BRISC complex subunit Abraxas 2	ABRAXAS2	upregulated in non-converter	0.72 [0.57-0.86]	76%	83%	68%	75%	77%	33.3	10.20	0.01
**P51451**	Tyrosine-protein kinase Blk	BLK	upregulated in non-converter	0.72 [0.58-0.87]	69%	78%	59%	66%	73%	56.6	5.09	0.02
**Q7Z6M3**	Allergin-1	MILR1	upregulated in non-converter	0.73 [0.59-0.87]	72%	76%	67%	78%	64%	100.7	6.25	0.01
**P16152**	Carbonyl reductase [NADPH] 1	CBR1	upregulated in non-converter	0.77 [0.63-0.9]	70%	75%	64%	75%	64%	1917.3	5.25	0.01
**Q9HBG7**	T-lymphocyte surface antigen Ly-9	LY9	upregulated in converter	0.76 [0.63-0.9]	70%	75%	64%	75%	64%	169.5	5.25	0.01
**Q14129**	Protein DGCR6	DGCR6	upregulated in converter	0.74 [0.6-0.88]	72%	79%	64%	72%	73%	94.9	6.81	0.01
**P08476/P09529**	Inhibin beta A/B chain	INHBA/INHBB	upregulated in non-converter	0.75 [0.62-0.89]	74%	78%	68%	78%	68%	440.1	7.65	0.03
**P48745**	CCN family member 3	CCN3	upregulated in non-converter	0.73 [0.59-0.87]	72%	77%	65%	75%	68%	4284.3	6.43	0.02
**Q96IQ7**	V-set and immunoglobulin domain-containing protein 2	VSIG2	upregulated in non-converter	0.73 [0.58-0.87]	70%	77%	63%	72%	68%	246.5	5.48	0.01
**Q13356**	RING-type E3 ubiquitin-protein ligase PPIL2	PPIL2	upregulated in converter	0.66 [0.51-0.81]	72%	74%	68%	81%	59%	94.1	6.26	0.03
**Q99969**	Retinoic acid receptor responder protein 2	RARRES2	upregulated in non-converter	0.73 [0.59-0.87]	74%	73%	79%	91%	50%	4102.7	9.67	0.01

**Q9H4I9**	Essential MCU regulator, mitochondrial	SMDT1	upregulated in non-converter	0.78 [0.65-0.91]	74%	73%	79%	91%	50%	23	9.67	0.02
**Q16585**	Beta-sarcoglycan	SGCB	upregulated in converter	0.77 [0.64-0.9]	72%	72%	73%	88%	50%	110.2	7.00	0.01
**P09341**	Growth-regulated alpha protein	CXCL1	upregulated in converter	0.73 [0.58-0.87]	72%	70%	82%	94%	41%	1289.6	10.38	0.03
**P12314**	High affinity immunoglobulin gamma Fc receptor I	FCGR1A	upregulated in non-converter	0.78 [0.65-0.91]	70%	79%	62%	69%	73%	181.2	5.87	0.01
**P12110**	Collagen alpha-2(VI) chain	COL6A2	upregulated in converter	0.67 [0.52-0.82]	72%	74%	68%	81%	59%	28.8	6.26	0.01
**Q76M96**	Coiled-coil domain-containing protein 80	CCDC80	upregulated in non-converter	0.71 [0.56-0.85]	72%	73%	71%	84%	55%	293.1	6.48	0.03
**O15068**	Guanine nucleotide exchange factor DBS	MCF2L	upregulated in converter	0.71 [0.56-0.85]	69%	71%	63%	78%	55%	100.4	4.29	0.02
**P45452**	Collagenase 3	MMP13	upregulated in non-converter	0.72 [0.57-0.86]	74%	70%	90%	97%	41%	93.5	21.46	0.02
**Q1W4C9**	Serine protease inhibitor Kazal-type 13	SPINK13	upregulated in non-converter	0.62 [0.46-0.77]	63%	88%	53%	44%	91%	63.6	7.78	0.02
**P28698**	Myeloid zinc finger 1	MZF1	upregulated in converter	0.74 [0.59-0.88]	74%	75%	72%	84%	59%	187.3	7.80	0.03
**O75071**	EF-hand calcium-binding domain-containing protein 14	EFCAB14	upregulated in non-converter	0.75 [0.62-0.89]	61%	100%	51%	34%	100%	857.1	23.05	0.03

**Q9BWV2**	Spermatogenesis-associated protein 9	SPATA9	upregulated in non-converter	0.73 [0.59-0.87]	63%	83%	53%	47%	86%	235.6	5.59	0.02
**Q92583**	C-C motif chemokine 17	CCL17	upregulated in converter	0.68 [0.53-0.83]	76%	73%	85%	94%	50%	106	15.00	0.03
**O94923**	D-glucuronyl C5-epimerase	GLCE	upregulated in non-converter	0.56 [0.41-0.72]	61%	92%	51%	38%	95%	495.1	12.60	0.04
**Q9UJU6**	Drebrin-like protein	DBNL	upregulated in non-converter	0.66 [0.51-0.81]	67%	82%	56%	56%	82%	379.5	5.79	0.03
**A6NNA5**	Dorsal root ganglia homeobox protein	DRGX	upregulated in non-converter	0.59 [0.44-0.75]	69%	78%	59%	66%	73%	95.7	5.09	0.01
**P61978**	Heterogeneous nuclear ribonucleoprotein K	HNRNPK	upregulated in non-converter	0.57 [0.41-0.73]	67%	73%	58%	69%	64%	55.3	3.85	0.06
**O15146**	Muscle, skeletal receptor tyrosine-protein kinase	MUSK	upregulated in non-converter	0.73 [0.59-0.87]	74%	70%	100%	100%	36%	118.1	36.57	0.02
**P16035**	Metalloproteinase inhibitor 2	TIMP2	upregulated in non-converter	0.63 [0.47-0.78]	67%	85%	56%	53%	86%	1771.4	7.18	0.02
**Q9UN72**	Protocadherin alpha-7	PCDHA7	upregulated in non-converter	0.68 [0.53-0.83]	70%	77%	63%	72%	68%	450.2	5.48	0.04
**Q9BS26**	Endoplasmic reticulum resident protein 44	ERP44	upregulated in non-converter	0.67 [0.52-0.82]	65%	78%	55%	56%	77%	548.4	4.37	0.04
**Q9UBU2**	Dickkopf-related protein 2	DKK2	upregulated in non-converter	0.64 [0.48-0.79]	67%	71%	60%	75%	55%	1302.6	3.60	0.04

**P35443**	Thrombospondin-4	THBS4	upregulated in non-converter	0.68 [0.53-0.83]	70%	69%	75%	91%	41%	229.3	6.69	0.03
**P29965**	CD40 ligand	CD40LG	upregulated in non-converter	0.65 [0.5-0.8]	61%	87%	51%	41%	91%	58.3	6.84	0.06
**O86476**	OS=Staphylococcus aureus (strain Newman) OX=426430 GN=clfB PE=1 SV=2	None	upregulated in converter	0.66 [0.5-0.81]	65%	76%	55%	59%	73%	167.3	3.90	0.07
**P04049**	RAF proto-oncogene serine	RAF1	upregulated in non-converter	0.65 [0.5-0.8]	65%	74%	56%	63%	68%	202.2	3.57	0.08
**Q9BY71**	Leucine-rich repeat-containing protein 3	LRRC3	upregulated in non-converter	0.73 [0.58-0.87]	67%	82%	56%	56%	82%	125.4	5.79	0.08
**P22888**	Lutropin-choriogonadotropic hormone receptor	LHCGR	upregulated in non-converter	0.69 [0.55-0.84]	67%	77%	57%	63%	73%	48.4	4.44	0.13
**P14136**	Glial fibrillary acidic protein	GFAP	upregulated in converter	0.7 [0.56-0.85]	69%	73%	62%	75%	59%	9851.5	4.33	0.13
**P08833**	Insulin-like growth factor-binding protein 1	IGFBP1	upregulated in non-converter	0.55 [0.39-0.71]	67%	68%	63%	81%	45%	54.7	3.61	0.1
**Q14457**	Beclin-1	BECN1	upregulated in converter	0.65 [0.5-0.81]	33%	36%	33%	16%	59%	75.7	0.27	0.21
**Q01968**	Inositol polyphosphate 5-phosphatase OCRL-1	OCRL	upregulated in non-converter	0.68 [0.53-0.83]	67%	69%	61%	78%	50%	218.3	3.57	0.3
**P10914**	Interferon regulatory factor 1	IRF1	upregulated in non-converter	0.69 [0.54-0.84]	67%	72%	59%	72%	59%	63	3.69	0.33

**P19419**	ETS domain-containing protein Elk-1	ELK1	upregulated in converter	0.48 [0.32-0.64]	61%	67%	52%	69%	50%	147.2	2.20	0.36
**P51814**	Zinc finger protein 41	ZNF41	upregulated in non-converter	0.52 [0.36-0.68]	56%	68%	47%	47%	68%	83.5	1.89	0.53
**P43320**	Beta-crystallin B2	CRYBB2	upregulated in non-converter	0.46 [0.31-0.62]	59%	67%	50%	63%	55%	139.4	2.00	0.76
**P43080**	Guanylyl cyclase-activating protein 1	GUCA1A	upregulated in non-converter	0.59 [0.43-0.74]	46%	56%	38%	44%	50%	78.7	0.78	0.88

Supplementary Table 2. Predictive performance of the top 20 identified CSF protein biomarkers with highest sensitivity compared to the performance of OCGB status. Proteins are listed from highest to lowest sensitivity. Abbreviations: AUC, area under the curve; CI, confidence interval, Acc, Accuracy; Sens, Sensitivity; Spec, specificity; PPV, positive predictive value; NPV, negative predictive value; ROC, receiver operator curve.

	**AUC [95% CI]**	**Acc**	**Sens**	**Spec**	**PPV**	**NPV**	**ROC threshold**	**Odds Ratio**	**P-value**
**TPM3**	0.69 [0.54-0.84]	78%	100%	63%	65%	100%	150.3	73.33	0.001
**EFCAB14**	0.75 [0.62-0.89]	61%	100%	34%	51%	100%	857.1	23.05	0.03
**OCGB status**	0.66 [0.5-0.81]	59%	100%	31%	50%	100%	0.5	21.00	0.01
**GLCE**	0.56 [0.41-0.72]	0.61	0.95	0.38	0.51	0.92	495.1	12.60	0.04
**DYNLT1**	0.83 [0.71-0.95]	78%	91%	69%	67%	92%	104.5	22.00	<0.001
**NCR1**	0.79 [0.66-0.92]	80%	91%	72%	69%	92%	205.5	25.56	<0.001
**ADRM1**	0.74 [0.6-0.88]	72%	91%	59%	61%	90%	65.9	14.62	0.003
**MAVS**	0.73 [0.59-0.87]	70%	91%	56%	59%	90%	58.2	12.86	0.002
**ACOT7**	0.78 [0.65-0.91]	74%	91%	63%	63%	91%	109.2	16.67	0.002
**NR1D2**	0.68	72%	91%	59%	61%	90%	53.3	14.62	0.004

	[0.54-0.83]								
**IL32**	0.72 [0.57-0.86]	72%	91%	59%	61%	90%	67	14.62	0.004
**CFH**	0.78 [0.65-0.91]	72%	91%	59%	61%	90%	4803.9	14.62	0.01
**ELL2**	0.79 [0.66-0.92]	69%	91%	53%	57%	89%	279.8	11.33	0.01
**SPINK13**	0.62 [0.46-0.77]	63%	91%	44%	53%	88%	63.6	7.78	0.02
**CD40LG**	0.65 [0.5-0.8]	61%	91%	41%	51%	87%	58.3	6.84	0.06
**GPNMB**	0.76 [0.62-0.9]	65%	86%	50%	54%	84%	616.9	6.33	0.02
**SPATA9**	0.73 [0.59-0.87]	63%	86%	47%	53%	83%	235.6	5.59	0.02
**TIMP2**	0.63 [0.47-0.78]	67%	86%	53%	56%	85%	1771.4	7.18	0.02
**GCH1**	0.74 [0.6-0.88]	76%	82%	72%	67%	85%	85.3	11.50	0.003
**EDAR**	0.72 [0.58-0.86]	72%	82%	66%	62%	84%	428.6	8.59	0.003
**CTSK**	0.74 [0.6-0.88]	72%	82%	66%	62%	84%	257.1	8.59	0.01
**PTGR1**	0.75 [0.61-0.89]	74%	82%	69%	64%	85%	175.5	9.90	0.002

Detailed statistical methods.

Stage 1. Model validation

OPLS-DA models were optimized by internal 7-fold cross-validation. The quality of classification was assessed using a 10-fold external cross-validation scheme with 1000 repetitions in total, correcting for unequal class sizes. This validation scheme involves multiple iterations of splitting the data into training and testing sets, which ensures that any discrimination observed in the models cannot have occurred by chance. The training data is used to estimate the model parameters (number of components, R^2 – the 'goodness' of fit, and Q^2 – the internal predictive performance of the model on the training dataset) and learn the underlying discriminatory patterns between the groups under consideration, whereas the independent test set is employed to assess the accuracy, sensitivity, specificity, and generalizability of the trained models in the ensemble. We quantified the outcome of the cross validation by calculating the accuracy, sensitivity, and specificity of each model (n = 1000) from the predicted classifications of the external independent test set, which was not used to build each model. It is important to appreciate that the classifier (OPLS-DA) was blinded to each test set when training each model. This validation scheme tends to avoid over-fitting and helps assess the generalizability of the model to previously unseen datasets. For an exhaustive discussion on validation of this approach see Arlot and Celisse (2010). These values were compared with those of a null distribution (obtained from randomly permuting the classifications) using the two-sided Kolmogorov-Smirnov test (significant if p-value 0.05 or less).

Stage 2. Prediction and identification of discriminatory metabolites.

If stage 1 of the analysis reveals that the OPLS-DA classifier performs significantly better than chance, the separation observed between classes is confirmed and it is valid to interrogate metabolites driving the separation and to use the data for prediction of additional samples. Discriminatory variables were identified by calculating the average of the variable importance (VIP) scores of the ensemble of models. A VIP cut-off of 1.5 was used to identify the most important variables driving the separation between classes. Two-sample t-tests and ROC analysis was applied to each of the variables identified as discriminatory by the multivariate analysis to investigate the diagnostic accuracy of each biomarker in isolation. The p-values obtained were then corrected for multiple comparisons using the Bonferroni correction.
